# Parallel development of object recognition in newborn chicks and deep neural networks

**DOI:** 10.1371/journal.pcbi.1012600

**Published:** 2024-12-02

**Authors:** Lalit Pandey, Donsuk Lee, Samantha M. W. Wood, Justin N. Wood

**Affiliations:** 1 Informatics Department, Indiana University, Bloomington, Indiana, United States of America; 2 Cognitive Science Program, Indiana University, Bloomington, Indiana, United States of America; 3 Department of Neuroscience, Indiana University, Bloomington, Indiana, United States of America; 4 Center for the Integrated Study of Animal Behavior, Indiana University, Bloomington, Indiana, United States of America; Chinese Academy of Sciences, CHINA

## Abstract

How do newborns learn to see? We propose that visual systems are space-time fitters, meaning visual development can be understood as a blind fitting process (akin to evolution) in which visual systems gradually adapt to the spatiotemporal data distributions in the newborn’s environment. To test whether space-time fitting is a viable theory for learning how to see, we performed parallel controlled-rearing experiments on newborn chicks and deep neural networks (DNNs), including CNNs and transformers. First, we raised newborn chicks in impoverished environments containing a single object, then simulated those environments in a video game engine. Second, we recorded first-person images from agents moving through the virtual animal chambers and used those images to train DNNs. Third, we compared the viewpoint-invariant object recognition performance of the chicks and DNNs. When DNNs received the same visual diet (training data) as chicks, the models developed common object recognition skills as chicks. DNNs that used time as a teaching signal—space-time fitters—also showed common patterns of successes and failures across the test viewpoints as chicks. Thus, DNNs can learn object recognition in the same impoverished environments as newborn animals. We argue that space-time fitters can serve as formal scientific models of newborn visual systems, providing image-computable models for studying how newborns learn to see from raw visual experiences.

## Author summary

Do machines learn like brains? The performance of all learning systems depends on both the learning machinery and experiences (training data) from which the system learns, so answering this question will require giving machines and brains the same training data. To do so, we introduce a digital twin method for running parallel controlled-rearing studies on newborn animals and deep neural networks. We show that when deep neural networks (CNNs and transformers) are trained in the same visual environments as newborn chicks, the models develop the same object recognition skills as chicks. Both newborn chicks and deep neural networks can learn invariant object representations that generalize across novel viewpoints, even when learning occurs in an impoverished environment containing a single object seen from a limited 60° viewpoint range. Our study shows that blind fitting processes (variation + selection learning) can mimic the rapid visual learning of precocial newborn animals, in the absence of innate (hardcoded) knowledge about objects or space. We argue that visual development can be understood as space-time fitting, in which visual systems gradually adapt to the spatiotemporal data distributions in the environment.

## Introduction

How do newborns learn to see and understand the world? This question has inspired philosophers and scientists for more than 2,000 years, leading to vigorous debate regarding the core learning mechanisms in brains and the role of experience in shaping perceptual and cognitive skills. Nativist theorists have proposed that visual perception depends on innate (unlearned) primitives. For example, Gestalt psychologists argued that principles of visual organization are innate, with brains prewired to organize sensory input according to certain rules [1]; Biederman [2] argued that object perception depends on geometric primitives; and Spelke [3,4] argued that object perception depends on innate systems for object tracking and representation. A central assumption underlying nativist theories is that a newborn’s visual experiences are sparse, noisy, and impoverished. Given such ‘low quality’ training data, nativists argue that newborns need rich innate structures to learn how to see.

In contrast, empiricists argue that visual perception is learned. Evidence for visual learning comes from perceptual learning effects in human adults [5–10], studies of object learning in newborn animals [11–13], human infants [14,15], and children [16,17], and studies showing that visual perception substrates in brains are plastic [18,19]. According to one empiricist view—the direct-fit perspective [20]—learning processes can be understood through the same lens as evolutionary processes (see also refs. [21,22]). In evolution, genetic variation generates a range of possibilities, and selection filters those possibilities based on fitness. Likewise, learning can be conceptualized as a variation + selection process, in which prenatal development generates a range of variation (trillions of connection weights), and experience selects (strengthens/weakens) those connection weights to produce adaptive behavior. A central assumption underlying empiricist theories—including the direct-fit perspective—is that visual experience is structured across space and time, providing ‘high quality’ data for learning [23,24]. Thus, nativists and empiricists have different intuitions about whether the visual experiences available to newborns provide sufficient information for learning how to see.

How do we move beyond intuitions to formally characterize the core learning machinery in brains? One strategy is to build working artificial visual systems to test which learning machinery is sufficient in order to learn like newborn brains. Like biological visual systems, the artificial visual systems should be image computable (learn from raw visual inputs), so the biological and artificial systems are forced to solve the same learning problem. With this approach, theorists no longer need to rely on their intuitions about what is learnable and what is not [25]. Rather, they can run theoretical simulations testing whether particular learning mechanisms produce the same learning outcomes as newborn animals when given the same visual experiences as animals. Here, we use this reverse-engineering approach to directly test whether the visual experiences available to newborn animals are sufficient for direct-fit (empiricist) models to learn object recognition.

### Using machine learning algorithms as models of visual learning

To build working computational models of newborn visual systems, we used deep neural networks (DNNs). DNNs are direct-fit models because they use evolution-like (variation + selection) learning processes: the models start with random weights and large numbers of connections (variation), followed by gradual adjustment of those weights to optimize the learning objective (selection). Thus, DNNs learn through blind brute-force fitting, using local computations to discover task-relevant manifolds in a high-dimensional representational space [20]. In artificial intelligence, direct-fit models offer robust solutions for building visual intelligence [30–34]. Following Hasson, Nastase, & Goldstein [20], we suggest that direct-fit models can also be viable scientific hypotheses of how newborns learn to see.

After decades of lagging behind the recognition abilities of even young children, DNNs can now rival human adults on challenging object recognition tasks [26–29]. In addition to powering new technologies (e.g., autonomous driving, diagnosing radiation scans, automated face recognition), DNNs now serve as formal scientific models in psychology and neuroscience [30–34]. DNNs are valuable from a scientific perspective because they are image computable, allowing the models to generate specific neural and behavioral predictions on an image-by-image basis. The vast majority of studies using DNNs as models of the brain have compared DNNs to mature subjects; however, since DNNs are image computable, they can also serve as scientific models of visual learning.

Do DNNs produce the same learning outcomes as brains? When DNNs are trained ‘through the eyes’ of developing humans (i.e., by using head-mounted camera data collected from young children), DNNs develop high neural prediction accuracy in multiple areas of the ventral visual system [35], while also developing visual skills (e.g., object recognition and segmentation) that resemble those in humans [35–37]. These studies suggest that DNNs can learn human-like visual abilities when trained with biologically plausible data (embodied visual data streams).

To date, however, it is unknown whether DNNs can learn object recognition under the conditions faced by newborns, when training data are sparse and impoverished. DNNs are widely assumed to require extensive training data to learn visual intelligence [38–41]. Conversely, newborn animals rapidly learn to solve challenging visual tasks, with many abilities emerging within the first few days of life [42–44]. Despite having no prior visual experience with the world, newborns can learn to solve challenging visual tasks when they encounter their first object [42].

Controlled-rearing studies—in which researchers control the experiences (training data) available to newborn animals—provide particularly striking examples of the power and efficiency of newborn vision [42,45,46]. For example, newborn chicks can rapidly learn to solve core object perception tasks, in the absence of extensive visual experience with objects. Soon after hatching, chicks can parse objects from backgrounds [47], bind colors and shapes into integrated object representations [48], recognize objects and faces across novel views [42,49–51], and remember objects that have moved out of view (object permanence) [52]. Chicks learn all of these abilities even when reared in impoverished environments containing a single object. From a machine-learning perspective, this seems like an impressive feat: DNNs are typically trained on millions of images from thousands of object categories, whereas newborn chicks develop reasonably accurate object representations from experience with a single object. There appears to be a mismatch between the amount of training data needed by newborn brains versus DNNs.

However, the learning gap between brains and DNNs may not be as large as it appears. The criticism that DNNs require more training data than brains rests on the assumption that the visual experiences available to newborn animals are sparse, noisy, and impoverished. But, research from developmental psychology suggests that this assumption is misleading. After capturing images from infants’ first-person perspective during natural everyday experience, researchers discovered that infants have access to embodied data streams with rich spatial and temporal structure [23]. By moving their bodies and interacting with objects, infants can rapidly acquire large numbers of diverse, high-quality object views that are well suited for visual learning [53,54]. Consequently, embodied visual experiences might provide ample opportunities for learning, even in the impoverished environments from controlled-rearing studies (e.g., environments with a single object). Rich, embodied data streams may help explain how newborn animals learn so rapidly and efficiently about the world.

**Fig 1:**
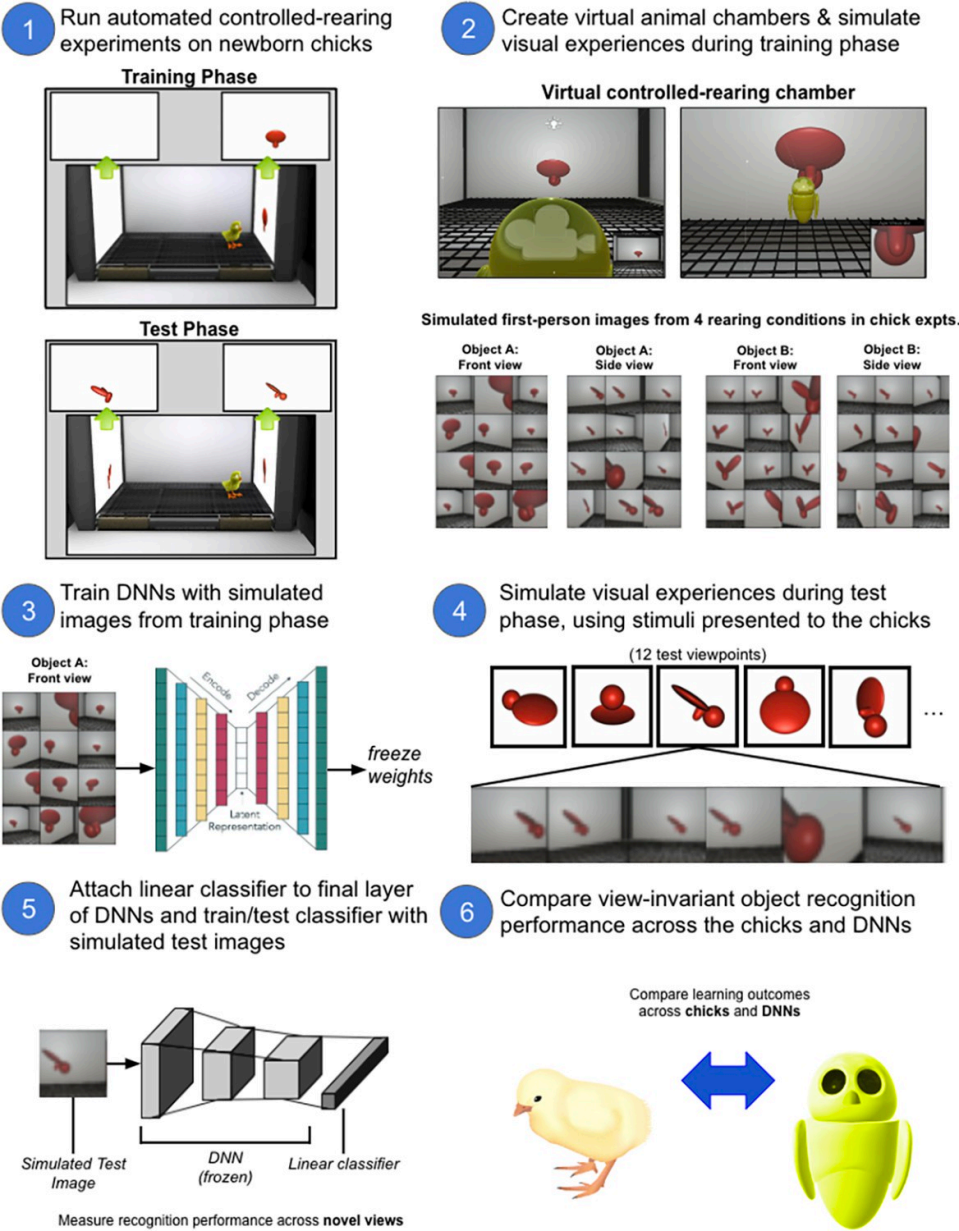
Digital Twin Method. **(1)** Run controlled-rearing experiments testing how view-invariant object recognition develops in newborn chicks. **(2)** Simulate the visual experiences available to the chicks during the training phase. **(3)** Train self-supervised DNNs with the simulated images, then freeze the DNN weights to prevent further learning. **(4)** Simulate the visual experiences available to the chicks during the test phase. **(5)** Evaluate the DNN’s view-invariant object recognition performance using a linear classifier, which is trained & tested with the simulated test images in a cross-validated design. **(6)** Compare the view-invariant recognition performance of the chicks and DNNs.

Ultimately, the performance of all learning systems depends on both the learning machinery and the experiences (training data) from which the system learns. Thus, determining whether DNNs learn like brains requires giving DNNs the same training data as brains. To do so, we performed parallel controlled-rearing experiments on newborn chicks and DNNs (Fig 1).

First, we raised newborn chicks in strictly controlled visual environments and measured the chicks’ object recognition performance [42]. Second, to simulate the training data available to the chicks, we created digital twins (virtual replicas) of the controlled-rearing chambers in a video game engine and recorded the first-person images acquired by agents moving through the chambers. Third, we trained self-supervised DNNs with the simulated first-person images from the virtual chambers, then tested the DNNs with the same images used to test the chicks. This digital twin method allows newborn chicks and DNNs to be trained and tested in the same visual environment, enabling direct comparison of their learning.

## Results

### Animal experiments & stimuli

We chose newborn chicks (*Gallus gallus*) as a model system because they are uniquely suited for studying the earliest stages of visual learning. First, unlike commonly used animal models in psychology and neuroscience (e.g., rodents, pigeons, monkeys), chickens are mobile on the first day of life and can be raised in strictly controlled environments from the onset of vision (e.g., environments without a caregiver). It is thus possible to control all of a chick’s visual training data. Second, it is possible to fully automate controlled-rearing studies of newborn chicks [55], producing data with a high signal-to-noise ratio. Third, avian and mammalian brains share many similarities [56–58]. On the circuit level, avian and mammalian brains have homologous cortical circuits for processing sensory input [58]. While these circuits are organized differently in birds and mammals (nuclear vs. layered organization, respectively), the circuits share similarities in terms of cell morphology, the connectivity pattern of the input and output neurons, gene expression, and function [57,59–61]. On the architectural level, avian and mammalian brains also share the same large-scale organizational principles: both are modular, small-world networks with a connective core of hub nodes that includes visual, auditory, limbic, prefrontal, premotor, and hippocampal structures [57]. If mammals and birds share homologous cortical circuits and architectures—as these studies suggest—then controlled-rearing studies of newborn chicks can inform our understanding of human visual development.

We focused on the behavioral results from Wood [42], which included data from 35 newborn chicks. In the study, chicks were hatched in darkness, then raised singly in automated controlled-rearing chambers that measured each chick’s behavior continuously (24/7) during the first two weeks of life (Fig 1, step 1). Each chamber had two display walls (LCD monitors) for displaying object stimuli. The chambers did not contain any objects other than the virtual objects projected on the display walls. Thus, the chambers provided full control over all of the visual object experiences available to the chicks from the onset of vision.

**Fig 2:**
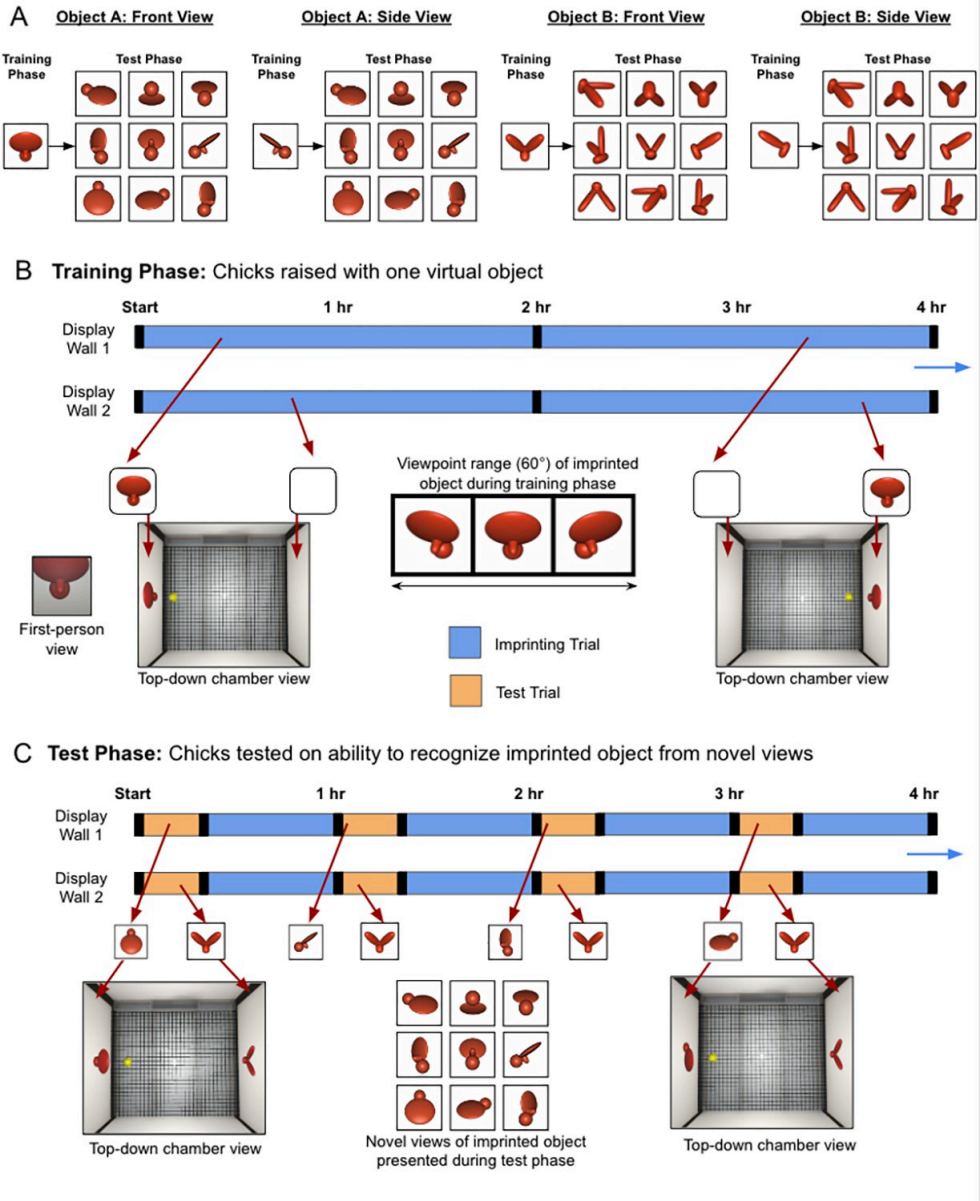
Chick Experiment. **(A)** The four train/test conditions presented to the chicks. **(B)** The schematic shows how the virtual objects were presented for sample 4-hr periods. During the training phase, a single virtual object appeared on one display wall at a time (indicated by blue segments on the timeline), switching walls every 2 hr, after a 1-min period of darkness (black segments). The object rotated back and forth through a limited 60° viewpoint range. **(C)** During the Test Phase, two virtual objects (one imprinted, the other novel) were shown simultaneously, one on each display wall, for 20 min per hour (orange segments). The illustrations below the timeline are examples of paired test objects displayed in four of the test trials. The test objects rotated through a 60° viewpoint range. Each test trial was followed by a 40-min rest period (blue segments). During the rest periods, the imprinting stimulus from the training phase was shown on one display wall, and the other display wall was blank. The illustrations show the displays seen by chicks that were imprinted to Object A: Front View (see Panel A).

The controlled-rearing study (Fig 2) used a similar design as machine-learning studies of DNNs. Specifically, the study was divided into a training phase and test phase. In the training phase, newborn chicks received a set of training data for learning (the visible features present in the controlled-rearing chamber). In the test phase, the automated chambers evaluated the chicks’ object recognition performance by presenting novel test images to the chicks (i.e., new views of the imprinted object not seen in the training phase, Fig 2A). Likewise, in machine-learning studies, researchers provide DNNs with a set of training data for learning (training phase), then freeze the weights and evaluate the DNN’s generalization performance across novel test images (test phase). This parallel experimental design across studies of newborn chicks and DNNs permits direct comparison of their learning outcomes.

During the training phase (first week, Fig 2B), Wood [42] reared newborn chicks in an environment containing a single 3D object rotating through a 60° viewpoint range. The object completed the full back and forth rotation every 6s. This virtual object was the only object in the chick’s environment. The chicks were raised in this environment for one week, allowing the critical period on filial imprinting (learning) to close before the test phase. The chicks were raised in one of four rearing environments (Fig 2A).

During the test phase (second week, Fig 2C), Wood [42] tested whether the chicks could recognize their imprinted object across 12 viewpoint ranges (11 novel, 1 familiar). These in-depth viewpoint changes introduced large, novel, and complex changes in the object’s appearance on the retina [62]. To test whether the chicks could recognize their imprinted object across the viewpoint changes, Wood used an automated two-alternative forced-choice procedure. On each test trial, the imprinted object appeared on one display wall and an unfamiliar object appeared on the opposite display wall. Test trials were scored as “correct” when the chicks spent a greater proportion of time with their imprinted object and “incorrect” when the chicks spent a greater proportion of time with the unfamiliar object. By leveraging automation, Wood [42] collected hundreds of test trials from each chick, producing data with a high signal-to-noise ratio.

The chicks performed well on the task, successfully recognizing their imprinted object from both the familiar view and the 11 novel views [42]. Despite being raised in impoverished environments containing a single object, the chicks developed view-invariant object recognition. Recognition performance was high even when the within-object image difference (i.e., the pixel-level difference between the test video of the imprinted object and the training video of the imprinted object) was greater than the between-object image difference (i.e., the pixel-level difference between the test video of the unfamiliar object and the training video of the imprinted object), which meets a reasonable operational definition of invariant object recognition [62–64]. These results suggest that newborn chicks can learn view-invariant object features in impoverished environments. Are DNNs capable of similar feats of visual learning?

### Experiment 1: Can direct-fit models learn object recognition in the same impoverished environments as newborn chicks?

To test whether direct-fit models can learn object recognition in impoverished environments, we used a class of DNN models called convolutional neural networks (CNNs). CNNs are directly inspired by neurophysiological observations of biological visual systems, including a restricted connectivity pattern that resembles the receptive field organization found in the animal visual cortex [65–67]. Like biological visual systems, CNNs can learn to recognize objects from high-dimensional sensory inputs without supervised reward signals [35,68–70], rivaling the behavioral performance of mature animals. CNNs can also produce internal unit response properties at each level of the network that are similar to actual neurophysiological responses at the corresponding levels in biological visual systems [71]. For instance, CNNs produce accurate predictions of image-evoked population responses across early, middle, and higher-level cortical visual areas in primates [31,35,72]. In addition to predicting neural responses, researchers can use CNNs to control neural activation by synthesizing novel images that elicit neural activation above naturally observed levels [73–75]. Thus, CNNs capture rich functional properties of how biological visual systems process information.

To explore whether CNNs can learn in the same environments as newborn chicks, we simulated the visual environment of the chicks in Wood [42], by creating realistic digital twins of the controlled-rearing chambers in a video game engine (Unity 3D; Fig 1, step 2). Then, we simulated the raw visual experiences available in a chick’s environment by recording the first-person images acquired by an agent moving through the virtual chamber. The agent moved forward and backward, turned left and right, and rotated its head along the three axes of rotation (yaw, pitch, roll). This simulation approach canvassed the range of visual experiences that chicks could acquire in the chamber. The approach did not directly simulate a specific chick’s visual experiences. The approach also did not capture views chicks may have seen of their own bodies (e.g., wings, feet). Our artificial agent could not see its body, so its visual diet was limited to views of the virtual chamber. As such, our simulation approach establishes a baseline of what is learnable when a model has access to the same visual environment as newborn chicks. As a starting point, we collected 10,000 first-person images from each of the four rearing conditions (Fig 2A) and used those images to train CNNs (Fig 1, step 3).

### Training the models

To train the CNNs, we used self-supervised learning algorithms. Self-supervised learning is the predominant way that biological systems learn: human infants start receiving labeled training data about objects only when they begin understanding language, and newborn animals receive little (if any) labeled training data during development. Modern self-supervised learning algorithms can perform well on object recognition tasks [35,68–70]. Some of these algorithms (e.g., contrastive embedding methods) can even achieve neural prediction accuracy in multiple areas of the ventral visual system that equals or exceeds supervised methods, following training on head-mounted camera data collected from children [35]. Self-supervised computer vision algorithms thus provide a promising starting point for building image-computable models of newborn visual systems.

We evaluated models from five popular self-supervised learning algorithms in computer vision: (1) autoencoders [76,77], (2) variational autoencoders [78,79], (3) SimCLR (a contrastive embedding method; 69), (4) BYOL (an asymmetric network method; [70]), and (5) Barlow Twins (a joint embedding learning method; [68]). We tested a range of self-supervised learning algorithms to explore whether any of these algorithms could learn object recognition in impoverished environments, and if so, whether the learning outcomes were unique to any particular algorithm or rather reflected general outcomes that emerge when CNNs learn from common visual experiences as newborn animals.

To enable direct comparison across the learning algorithms, we used the same architectural backbone, ResNet-18 [80], in all of the CNNs. None of the CNNs were pre-trained, and during training, the CNNs only received simulated training data from the virtual chambers. The chicks in Wood [42] were reared in one of four possible environments (Fig 2A), so we trained each CNN in a digital twin of one of these four environments. Thus, the CNNs and chicks had access to the same visual environment for learning object representations.

### Testing the models

To evaluate the features learned by the CNNs, we used linear classifiers. Linear classifiers are widely used in neuroscience research to quantify easily accessible, task-relevant information in a population of neurons. Accordingly, linear classifiers allow us to assess the representations learned by models during self-supervised learning. After each CNN was trained, we discarded all of the layers after the ResNet-18 backbone (e.g., decoder for autoencoders, projection head for SimCLR, online network for BYOL) and froze the weights in the network. We then evaluated the features in the CNN by adding a single fully connected linear classifier on top of the last layer of the backbone and training only the linear classifier on the object recognition task. If the CNNs learned linearly separable view-invariant object features, then the downstream linear classifier should successfully learn to recognize the objects across novel views.

We trained and tested the linear classifiers by simulating images from the test phase of the chick experiments and extracting the image features using the previously trained (and frozen) CNN. We collected test phase images by recording the first-person views acquired by an agent moving through the virtual chambers when the test stimuli were projected on the display walls (Fig 1, step 4). The test phase images simulated the first-person images available to the chicks during the test trials.

To evaluate the generalization capacities of the CNNs, we used a 12-fold cross-validated design. We divided the dataset into 12 folds, with each fold containing images of the imprinted object and the unfamiliar object rotating through one of the 12 viewpoint ranges presented to the chicks. Performance was evaluated by training the linear classifier (Fig 1, step 5) on the training set (11 folds = 11 viewpoint ranges) and then testing classification accuracy on the held-out test set (1 fold = 1 viewpoint range). We report average cross-validated performance on the held-out images not used to train the linear classifier. Thus, all of our results reflect the generalization performance of the CNNs across novel views.

The linear classifiers were trained using supervised learning, with the ground-truth labels (object identity) provided for each image. While supervised learning was not present in the chick experiments, linear classifiers are simply a formal way of quantifying the degree and form of learned representations in CNNs. In neuroscience, information that is available directly via a linear readout is generally considered to be explicitly represented by a model or brain region [81–84]. The linear classifier does not provide the CNN with new information but merely measures the relative placement of different images within the model’s existing feature space. Linear classifiers are also a reasonable approximation of downstream neural computation, since linear classifiers express a plausible rate-code model for downstream decoder neurons (i.e., linear weightings followed by a single threshold value; [85]).

### Model performance

**Fig 3:**
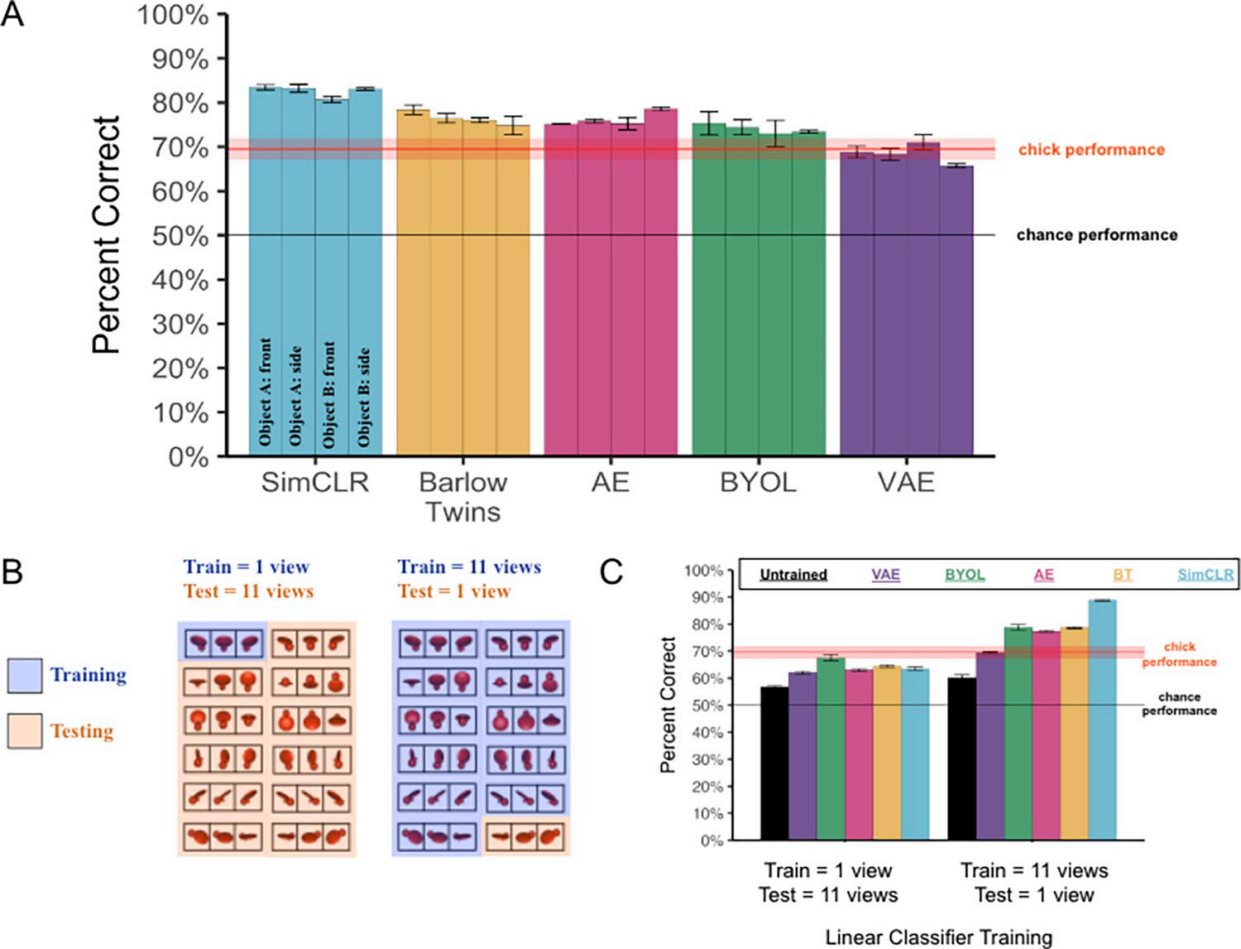
Experiment 1 Results. **(A)** View-invariant recognition performance of the five self-supervised CNN models across the four rearing conditions. The red horizontal line shows the chicks’ performance, with the ribbon representing standard error. **(B)** Linear classifier training. We evaluated the accessibility of the view-invariant features by training/testing linear classifiers on different numbers of viewpoint ranges. We used a cross-validated design, with different viewpoint ranges in the training (*blue*) versus test (*orange*) image sets. Thus, all results reflect the generalization performance of CNNs across novel views. **(C)** The models successfully recognized the object across novel views, even when the linear classifiers were trained on a single viewpoint range. Error bars represent standard error of model performances across validation folds.

Fig 3A shows the view-invariant object recognition performance of the CNNs across the four rearing conditions from the chick experiments. All of the learning algorithms performed above chance level (50%), scoring between 68.5% (VAE) and 82.6% (SimCLR) on the task (one-sample t-tests, all algorithms p < 10^−9^). Some learning algorithms were better than others at learning from embodied data streams in impoverished environments (Welch’s ANOVA, F(4, 27) = 66.9, p < 10^−12^). However, all of the learning algorithms succeeded across all four rearing conditions. These results demonstrate that self-supervised CNNs can spontaneously learn view-invariant object features in the same impoverished environments as newborn chicks (i.e., environments containing a single object seen from a limited 60° viewpoint range). Direct-fit models may thus be promising models for studying how newborns learn to see from raw visual experiences.

### Accessibility of view-invariant features

How accessible were the view-invariant features in the models? One possibility is that the view-invariant features were formatted inefficiently, such that a linear classifier would need large amounts of training views to learn the correct combination of features to solve this task. Alternatively, the view-invariant features might be formatted efficiently, allowing a linear classifier to learn the correct combination of features from just a handful of views. To distinguish between these possibilities, we again used a cross-validated design, but trained both the CNNs and linear classifiers on a single viewpoint range, then tested the CNNs on the held-out viewpoint ranges (train = 1; test = 11, Fig 3B). Training both the CNN and linear classifier on one viewpoint range more closely mimics the rearing conditions of the newborn chicks, since the chicks only saw one viewpoint range during the training phase. We then compared this linear classifier performance to the performance of the linear classifiers that were trained using 11 viewpoint ranges (train = 11; test = 1). If the view-invariant features were formatted efficiently, then the CNNs should perform reasonably well, even when the linear classifiers were trained on a single viewpoint range.

As expected, when the linear classifiers were trained on one viewpoint range, performance was lower than when the linear classifiers were trained on 11 viewpoint ranges (Fig 3C; paired t-tests, for each learning algorithm, all Ps < 10^−5^). Importantly, however, the linear classifiers still performed well above chance level even when trained on a single viewpoint range (one-sample t-tests for each learning algorithm, all Ps <10^−7^). Thus, when self-supervised CNNs are trained in the same visual environments as chicks, the CNNs learn linearly separable and accessible view-invariant object features.

### Learning without backpropagation

One limitation is that the models described above were trained with backpropagation, which is not biologically plausible as a learning mechanism for the brain [86–88]. Prior studies suggest that the brain does not have a global representation of error; rather, it makes predictions using local computations [89–91]. One hypothesis is that feedback connections in the brain produce neural activity that locally approximates backpropagation signals [87]. If so, then models that use local learning rules—rather than backpropagation—should still learn effectively when trained in the same visual environments as newborn chicks.

**Fig 4:**
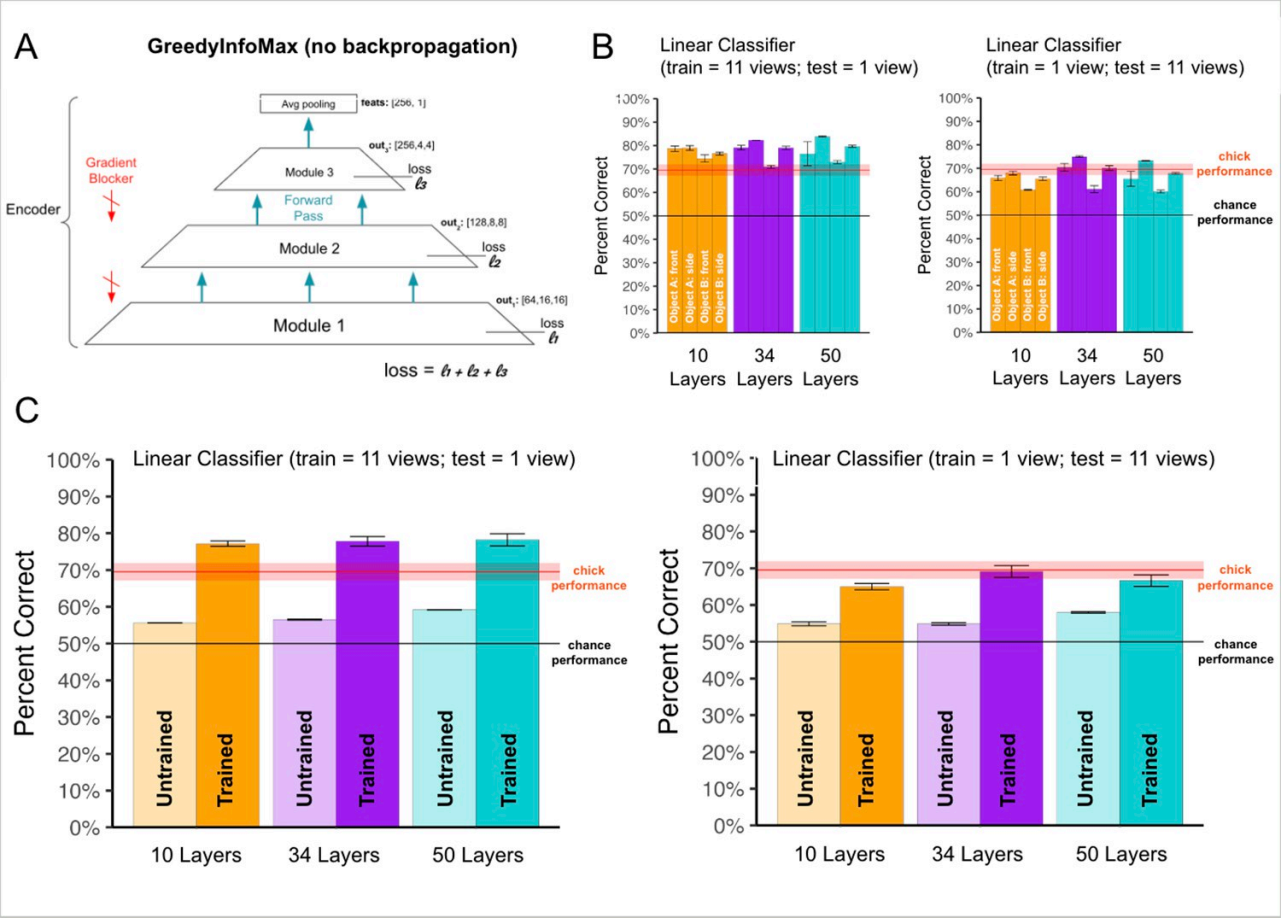
**(A)** Architecture of GreedyInfoMax (GIM) model. The CNN is divided into separate gradient-isolated modules, each with its own contrastive loss function. A gradient blocker blocks the backward flow of gradients, preventing backpropagation. The loss is calculated by taking the sum of individual losses within each module. **(B)** View-invariant recognition performance of newborn chicks and different GIM architecture sizes, across the four rearing conditions presented to the chicks. The red horizontal line shows the chicks’ performance. **(C)** Comparison of untrained versus trained GIM models across the three architecture sizes. All GIM models showed large learning gains, showing that CNNs without backpropagation can learn view-invariant features in the impoverished environments faced by newborn chicks. Error bars represent standard error of model performances across validation folds.

Some researchers argue that layers in the brain learn by optimally preserving input information before passing signals to the next layer, thus reducing prediction error [92]. Inspired by this idea, we tested the GreedyInfoMax (GIM) model [92], which learns by optimally maintaining mutual information across layers (Fig 4A). The GIM model uses a CNN backbone of convolutional layers and residual blocks. Each residual block functions as a gradient isolated module. Each module has a local contrastive loss objective (rather than a global objective). The GIM model deviates from traditional CNNs by blocking the backward flow of gradients, thereby preventing backpropagation.

When trained in the same visual environments as newborn chicks, the GIM model performed well above chance level on the view-invariant recognition task, across a range of architecture sizes (Fig 4B; one-sample t-tests, 10 layers: t(11) = 37.3, p < 10^−12^; 34 layers: t(11) = 21.4, p <10^−9^; 50 layers: t(11) = 17.2, p < 10^−8^). The GIM model also performed well when the linear classifier was trained on just a single viewpoint range, matching the chicks (one-sample t-tests, 10 layers: t(11) = 17.6, p < 10^−8^; 34 layers: t(11) = 11.9, p < 10^−6^; 50 layers: t(11) = 10.5, p < 10^−6^).

To quantify the impact of learning, we compared untrained to trained GIM models (Fig 4C). The trained GIM models showed large learning gains, matching chick-level performance, even when the linear classifier was trained on a single viewpoint range (paired two-sample t-tests comparing trained GIM to untrained performance, 10 layers: t(11) = 29.6, p < 10^−11^; 34 layers: t(11.3) = 16.3, p < 10^−8^; 50 layers: t(11.0) = 11.6, p < 10^−6^). Thus, CNNs can learn effectively in the impoverished visual environments faced by newborn chicks, even without backpropagation.

### Experiment 2: How do hardwired knowledge versus learning contribute to direct-fit models?

In principle, visual systems could fit to the environment through two processes: 1) evolutionary processes that hardcode knowledge through strong inductive biases and 2) learning processes. Formulated as models of adult visual systems, CNNs have a form of hardcoded visual knowledge reflecting the spatial structure of natural images, including local connectivity, parameter sharing, and hierarchical structure. This hardcoded ‘spatial knowledge’ allows CNNs to generalize well from small datasets and learn useful feature hierarchies that capture the structure of visual images.

### Contribution of hardcoded spatial knowledge

If the CNNs in Experiment 1 fit to the environments largely through hardcoded processes that assume the spatial structure of natural images, then untrained CNNs should perform as well as trained CNNs on our task. Moveover, untrained CNNs that have more hardcoded spatial operations (i.e., larger models with more layers) should perform better than untrained CNNs that have fewer spatial operations (i.e., smaller models with fewer layers).

**Fig 5:**
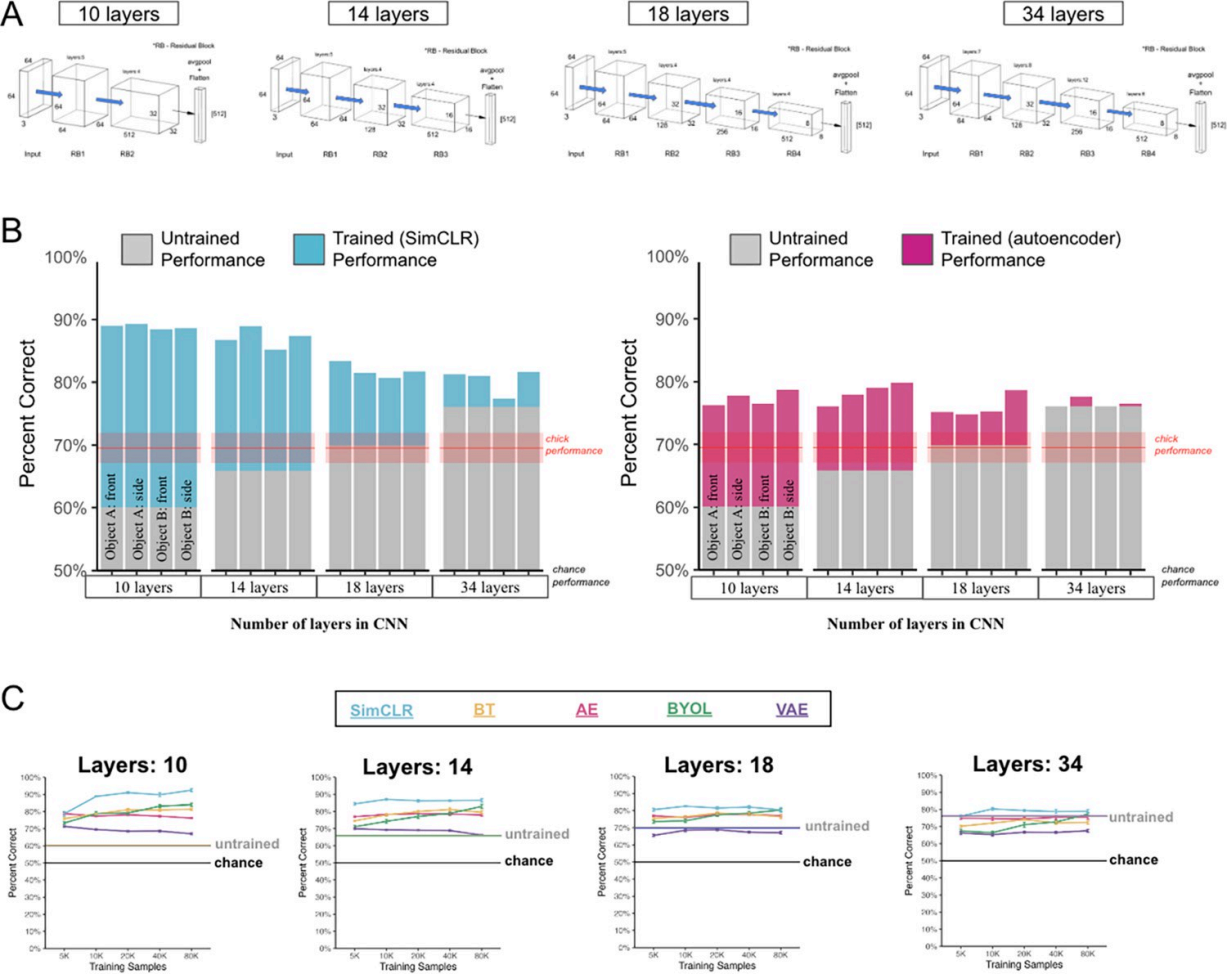
Experiment 2. **(A)** We increased the number of hardcoded spatial operations by adding more layers to the CNN architecture. To create different architecture sizes, we systematically added and removed residual blocks and bridge connections between blocks from the original ResNet architecture from Experiment 1. **(B)** Performance of the untrained CNNs (grey bars) increased when CNNs had more layers. Increasing the number of hardcoded spatial operations improved performance in untrained models. The colored bars show the learning gains that emerged in SimCLR (blue bars) and autoencoders (pink bars). Once the models were trained, performance decreased as a function of architecture size. Learning allowed smaller CNNs to achieve similar (or better) fits to the environment than larger CNNs, despite the smaller CNNs starting with weaker hardcoded spatial knowledge. Error bars represent standard error of model performances across validation folds. The red line shows the chicks’ performance, with the ribbon representing standard error. **(C)** To test whether learning plateaued in the models, we varied the number of images used to train the CNNs. Most models achieved similar performance when trained on 5,000 to 80,000 images, and a few algorithms (SimCLR, BYOL) showed modest performance gains with more training.

To test this prediction, we measured how the performance of untrained CNNs changes as a function of architecture size. We created CNN architectures with different numbers of layers (10, 14, 18, 34 layers, Fig 5A), then tested whether a downstream linear classifier could learn to use the features from the untrained CNNs to solve the view-invariant recognition task.

All of the untrained CNNs (gray bars in Fig 5B) performed significantly above chance level (one-sample t-tests for each architecture size, all Ps < .05), with performance increasing systematically as a function of architecture size (Pearson correlation between number of CNN layers and performance, r(10) = .91, p = .00003). The smallest (10-layer) CNN scored 60.1% on the task, and the largest (34-layer) CNN scored 76.1%. This pattern shows that architecture alone (hardcoded spatial knowledge) can be an important contributor to object recognition performance. From a direct-fit perspective, increasing the number of hardcoded spatial operations improves performance in untrained models because greater amounts of spatial fit are hardcoded into the model.

### Contribution of learning

How does learning contribute to performance, above and beyond the hardcoded contribution provided by the architecture? The direct-fit view of learning makes a specific prediction. Smaller CNNs (with less hardcoded spatial knowledge) need to learn more to fit to the environment, whereas larger CNNs (with more hardcoded knowledge) need to learn less to achieve comparable fits. Thus, smaller CNNs should learn more than larger CNNs.

To test these predictions, we measured whether learning improves recognition performance across the four architecture sizes (colored bars in Fig 5B). Nearly all of the CNNs benefited from learning (compared to untrained CNN performance). As predicted, there was a strong negative relationship between the size of the learning gain and the architecture size, with smaller architectures showing larger learning gains than larger architectures. (For all algorithms, we computed the Pearson correlation between number of CNN layers and performance for trained networks, all rs < -.45 and all Ps < .01). For example, the performance of the untrained 10-layer CNN was low (60.1%), but the performance of the trained 10-layer SimCLR model was higher than all other models (88.9%): a 28.8% improvement due to learning. Conversely, the 34-layer SimCLR model only improved 4.2% from learning. A similar pattern occurred in the other four learning algorithms, with larger learning gains emerging in smaller versus larger architectures. After training, all of the architecture sizes reached similar levels of performance, with the smaller architectures often achieving higher performance than the larger architectures. Learning allowed smaller CNNs to achieve similar (or better) fits to the environment than larger CNNs, despite the smaller CNNs starting with weaker hardcoded spatial knowledge.

One potential critique is that the larger architectures might have required more training data than the smaller architectures to reach maximum performance, thereby masking the true maximum performance levels of the larger architectures. To test this possibility, we varied the number of images used to train the CNNs, ranging from 5,000 to 80,000 images sampled from the virtual chambers. For most learning algorithms, recognition performance improved when CNNs were trained on larger numbers of images, before reaching an asymptote in performance (Fig 5C). The key finding—larger learning gains for smaller architectures—was observed across the different-sized training datasets, confirming that the tradeoff between hardcoded knowledge and learning did not occur simply because larger architectures needed more training data than smaller architectures. For direct-fit models, hardcoded knowledge and learning trade-off systematically with one another. The model fits to the underlying data distributions in the training environment, and fitting can occur either from hardcoded operations or from learning.

### Learning from dense sampling

**Fig 6:**
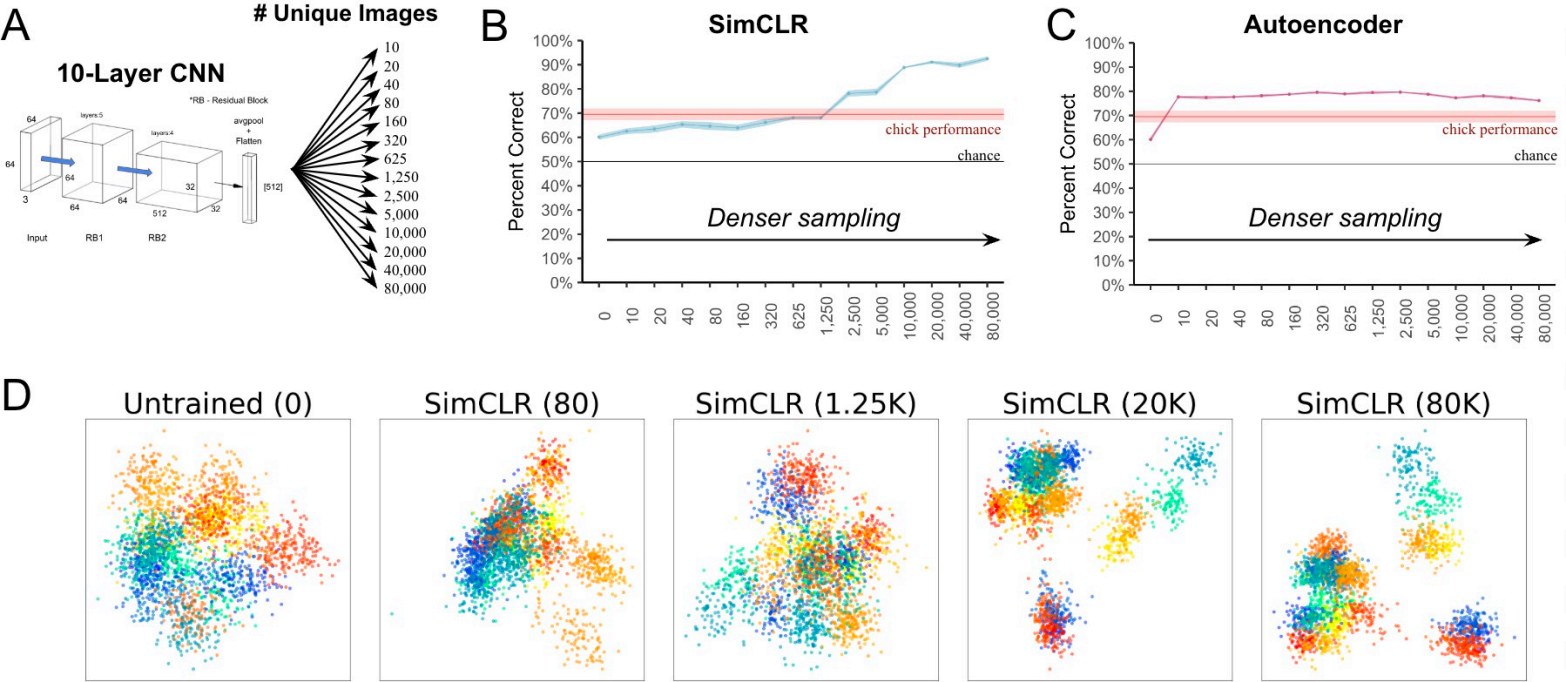
Measuring the impact of dense sampling of the visual environment on view-invariant recognition performance. **(A)** We trained the best performing CNN from the prior experiments (10-layer SimCLR) on datasets containing different numbers of images sampled from the virtual chamber. **(B)** Recognition performance improved systematically when the SimCLR model was trained on larger numbers of unique images. Denser sampling of the visual environment produced more accurate view-invariant object features. The red line shows the chicks’ performance, with the ribbon representing standard error. **(C)** The autoencoder algorithm did not achieve better performance when trained on larger numbers of images. This indicates that some learning algorithms (e.g., SimCLR) can leverage the unique views present in embodied data streams to build up accurate view-invariant features. **(D)** Two-dimensional projections of the feature representations from the untrained and trained CNNs. Each point represents a CNN representation of an input image containing a single object. Colors denote the identities and viewpoint ranges of the objects; warm colors (red-yellow) represent Object 1 and cold colors (green-purple) represent Object 2. Denser sampling of the visual environment led to more clustered representations in the embedding space.

Our simulations suggest a possible strategy newborns might use to learn how to see. By acquiring large numbers of views (retinal images) during visual exploration, newborn brains might gradually adapt (fit) to the underlying data distributions in the environment, without needing hardcoded knowledge of how proximal image features change as a function of distal object shape. To test whether CNNs can leverage this dense sampling strategy to learn object representations in impoverished environments, we selected the best performing model (SimCLR with 10 layers) and again manipulated the number of images used to train the models. We used a wider range of dataset sizes (0 to 80,000 images, Fig 6A) to more fully explore the impact of dense sampling on visual learning.

When the CNNs were trained on larger numbers of images, view-invariant recognition performance systematically increased, before reaching an asymptote in performance around 20,000 images (Fig 6B). The asymptote in performance reflects the model having achieved its best possible fit to the data distributions in the environment. For SimCLR, denser sampling of the visual environment led to more accurate representations of object shape, even in these simple environments containing a single object. Not all models showed this pattern. The autoencoder algorithm did not achieve better performance when trained on larger numbers of images (Fig 6C), indicating that some learning objectives are better than others at leveraging the unique images present in embodied data streams to build up view-invariant object features.

To visualize the impact of dense sampling on the representations learned by the SimCLR CNNs, we used linear discriminant analysis (LDA), creating two-dimensional projections of the feature representations from the final layer of the CNNs (Fig 6D). CNNs trained on larger numbers of images built more clustered representational spaces compared to untrained models.

**Fig 7:**
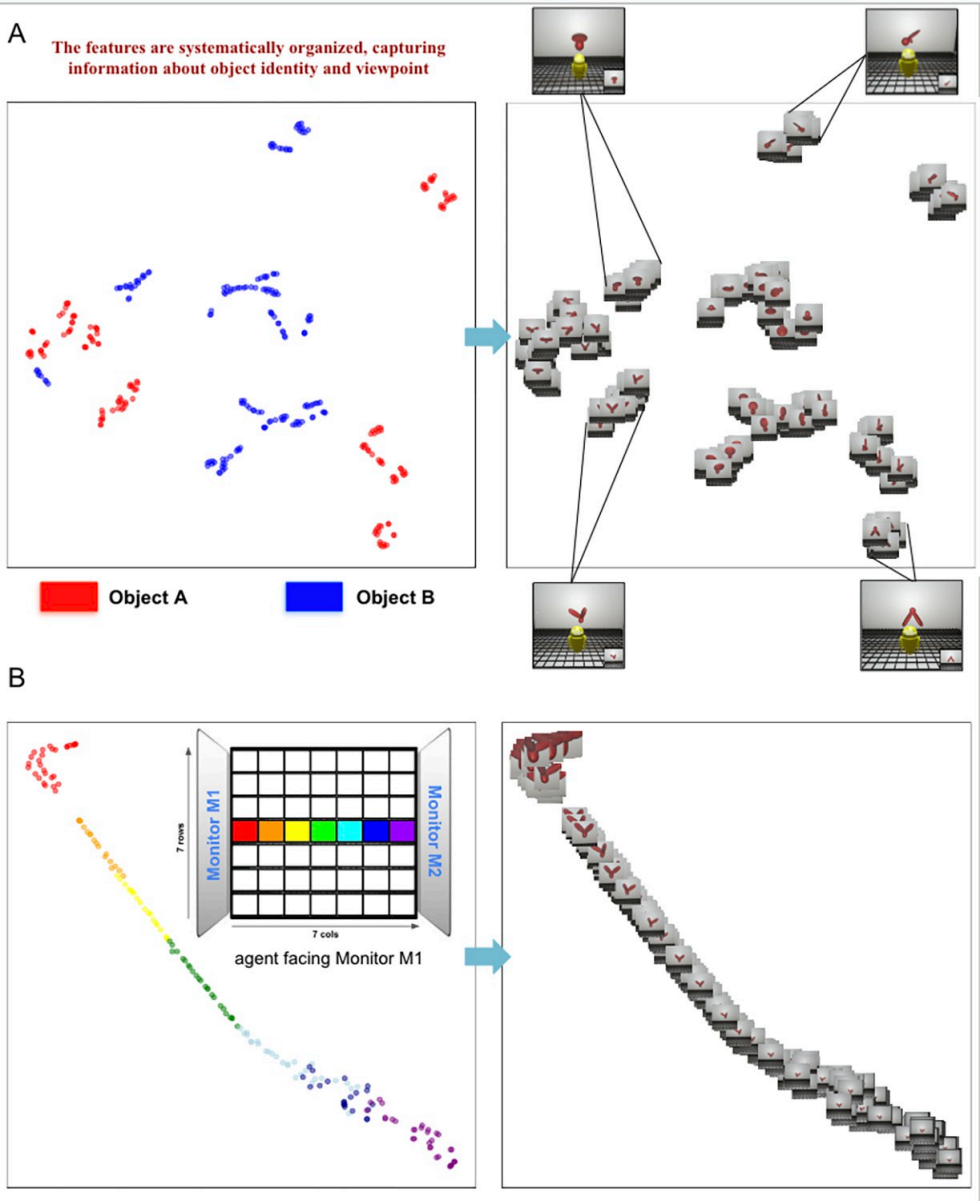
Visualizing the representations learned by models. t-SNE embeddings of the representations in the last layer of a CNN (10 layers, SimCLR algorithm, 80K training images). **(A)** For this visualization, the agent was stationary in front of the monitor while viewing Object A (red dots) or Object B (blue dots) from different test viewpoints. The CNN learned a structured feature space for representing both object identity and viewpoint. The images on the *right* correspond to the colored dots on the *left*. **(B)** For this visualization, the agent started at the front of the chamber (facing the object on Monitor M1), then moved straight backwards. The colored dots (*left*) denote the distance of the agent from the object. The images on the *right* correspond to the colored dots. The CNN learned a structured feature space for representing object distance and position in the chamber.

To visualize the features learned by the CNNs, we used t-distributed stochastic neighbor embedding (t-SNE), which does not require any supervised labels (Fig 7). The CNNs spontaneously learned a structured feature space for representing both object identity and viewpoint (Fig 7A). These results accord with findings that newborn chicks spontaneously encode information about both the identity and viewpoint of objects [49,50]. The CNNs also learned a structured feature space for representing object distance and position in the chamber (Fig 7B).

Taken together, Experiments 1–2 suggest that direct-fit learning via dense sampling is a viable strategy for learning how to see, even in the impoverished environments faced by newborn chicks. The first-person images obtained in these impoverished environments are sufficient for direct-fit models to learn view-invariant object features.

### Experiment 3: Can space-time fitting models learn like newborn chicks?

So far, all of the direct-fit models we tested learned from static images (e.g., by applying artificial image augmentations, like Gaussian blur and color jitter, or reconstructing individual images from a representational bottleneck). But, biological visual systems learn by exploiting the temporal structure of visual experience. There is extensive evidence that mature vision systems leverage time to build enduring object representations [5,8–10,18,19,93]. For example, when human adults see sequential views of an object, the views link together in memory, adapting to the spatiotemporal statistics of the visual environment [5–10]. Temporal learning effects have also been found on the neurophysiological level in adult monkeys [18,19,93,94]. There is even evidence that newborn animals use time as a teaching signal [11,12,51,95].

In chicks, the development of object parsing [47], visual binding [48], view-invariant object recognition [12,13,96], face recognition [51], and object permanence [52] all require experience of objects moving slowly and smoothly over time. If an object moves too quickly when being encoded into memory, the resulting object representation is distorted in the direction of object motion, effectively breaking invariant object recognition [12,51]. Likewise, if an object moves non-smoothly when being encoded into memory, chicks fail to solve simple color and shape recognition tasks [11] and their object representations fail to generalize across new viewpoints and rotation speeds [12,96]. Thus, the development of object recognition requires slow and smooth visual experiences with objects, adhering to the spatiotemporal properties of objects in the real world.

The studies cited above indicate that temporal learning plays a key role in visual learning. In machine learning, a subset of direct-fit models—space-time fitters—can also learn from temporal data without supervision (e.g., SimCLR-CLTT [97], ViT-CoT [98], VideoMAEs [99]). Since space-time fitting models have biologically plausible temporal learning objectives, we see them as promising models for studying visual development.

**Fig 8:**
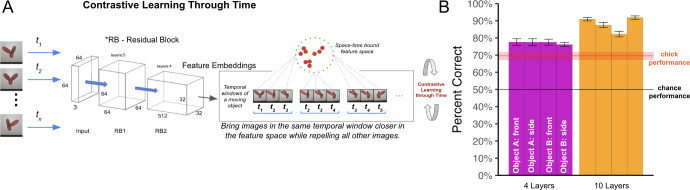
Experiment 3. **(A)** Contrastive Learning Through Time (CLTT) model. Each image is passed through a ResNet backbone, preserving the temporal order of images. Encoded features are aligned in the feature space using a temporal learning window of 3 frames. This window mimics the spike-timing-dependent plasticity learning window of biological visual systems (~300 ms). **(B)** View-invariant recognition performance of newborn chicks and SimCLR-CLTT models. We evaluated two architecture sizes (4-layer and 10-layer), across the four rearing conditions presented to the chicks. The red horizontal line shows the chicks’ performance. CNNs showed substantial learning gains over untrained CNN performance (untrained 4-layer CNN performance = 52.5%; untrained 10-layer CNN performance = 60.1%). CNNs can leverage time as a teaching signal to learn in impoverished environments. Error bars represent standard error of model performances across validation folds.

To explore whether space-time fitting models produce common learning outcomes as newborn chicks, we tested whether CNNs learn view-invariant object features when equipped with a temporal learning objective. We selected the best performing model from the previous experiments (SimCLR with 10 layers) and used a contrastive learning objective function that leverages the temporal structure of natural visual experience, without relying on artificial image augmentations [97]. The algorithm, Contrastive Learning Through Time (CLTT), contrasts temporally adjacent instances (positive examples) against non-adjacent instances (negative examples), to learn representations that capture underlying dynamics, context, and patterns across time (Fig 8A). The SimCLR-CLTT models used a temporal window of three frames to mimic the temporal learning window of biological visual systems (~300 milliseconds). This temporal duration corresponds to the observed neural firing period, which lasts between 100 to 400 milliseconds following image presentation [98, 102]. We used no artificial image augmentations in this experiment.

The SimCLR-CLTT models performed well above chance level on the view-invariant recognition task (one-sample t-test, t(11) = 30.3, p < 10^−11;^ Fig 8B). The models also performed above chance level when the linear classifier was trained on a single viewpoint range (one-sample t-test, t(11) = 13.1, p < 10^−7^). Like chicks, space-time fitting models can learn view-invariant object features in impoverished environments, by leveraging time as a teaching signal.

We also explored whether SimCLR-CLTT models can learn view-invariant features when equipped with a smaller architecture that does not provide strong hardcoded spatial knowledge. We built a 4-layer CLTT model and found that the untrained model performed near chance level (M = 52.5%). After training, the model scored 77.2% on the task. Thus, even small space-time fitting models can leverage time as a teaching signal to learn in impoverished environments. When models start with no initial ability to solve the task (chance-level untrained performance), they can still learn view-invariant features from embodied visual experiences with a single object.

### Experiment 4: Can generic space-time fitting models learn like newborn chicks?

The space-time fitting models in Experiment 3 learned view-invariant features in impoverished environments, but the models did have hardcoded spatial knowledge. As discussed in Experiment 2, CNNs have a hardcoded inductive bias reflecting the spatial structure of natural images. Conversely, more generic learning models—like vision transformers (ViTs)—do not have this hardcoded spatial knowledge. Rather, ViTs learn through flexible (learned) allocation of attention that does not assume any spatial structure. This flexibility allows ViTs to learn more abstract and generalizable features than CNNs, but it might also make ViTs less able to match the rapid learning abilities of newborn chicks.

**Fig 9:**
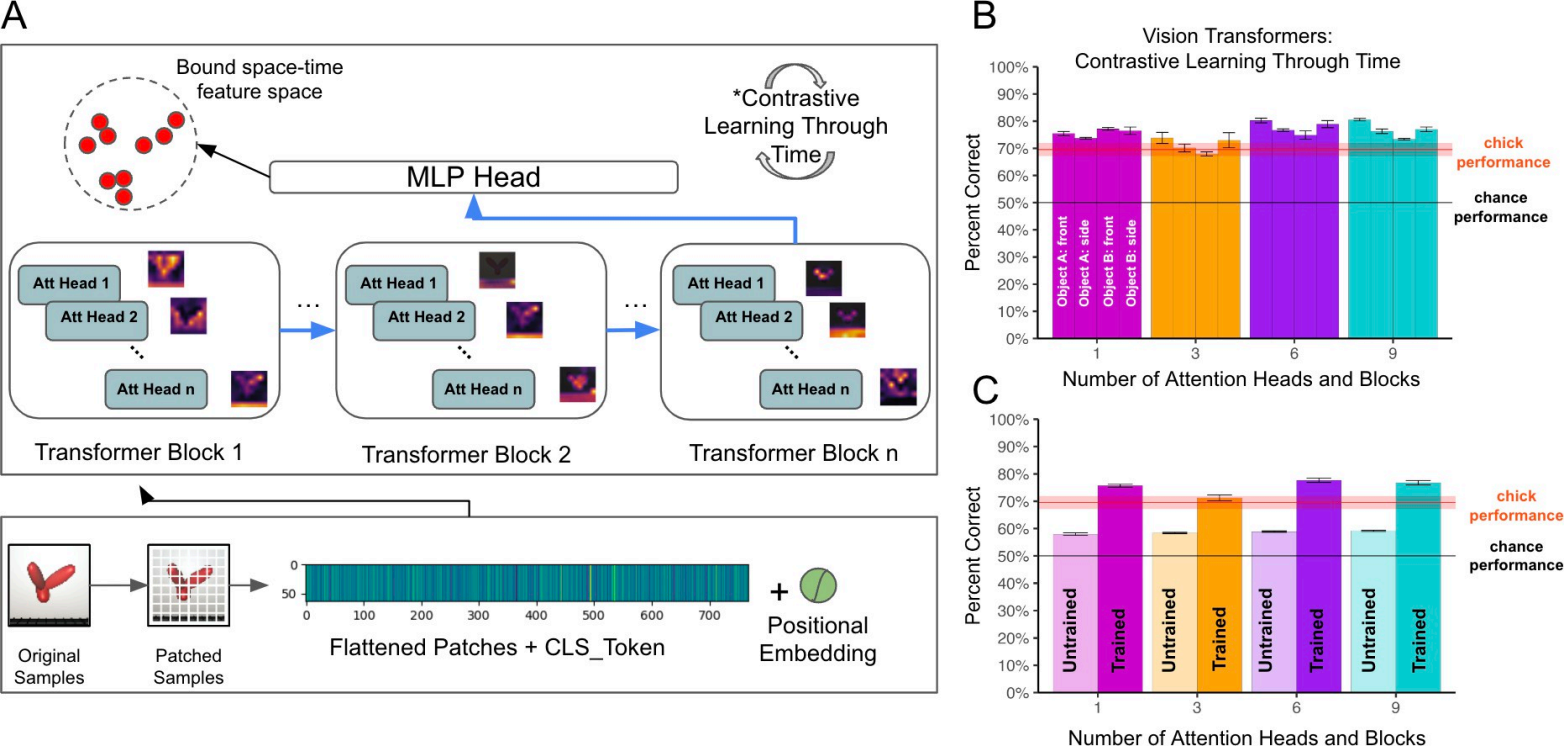
Experiment 4. **(A)** The vision transformer architecture. Images are first divided into smaller 8x8 patches and then reshaped into a sequence of flattened patches. A learnable positional embedding is added to each flattened patch, and a class token (CLS_Token) is added to the sequence. The resulting embedding is then sequentially processed by transformer blocks while also being analyzed in parallel by attention heads, which generate attention filters shown next to each head. The learned representation of the image is adjusted based on the contrastive learning through time loss function. **(B)** View-invariant recognition performance of newborn chicks and different ViT-CoT architecture sizes, across the four rearing conditions presented to the chicks. The red horizontal line shows the chicks’ performance. **(C)** Comparison of untrained versus trained ViT-CoT models across the four architecture sizes. All ViT-CoT models showed large learning gains, showing that vision transformers can learn view-invariant features in the impoverished environments faced by chicks. Error bars represent standard error of model performances across validation folds.

To test whether generic space-time fitting models can learn object recognition in the same impoverished environments as chicks, we modified the CLTT algorithm from Experiment 3, replacing the CNN with a ViT backbone [98]. This self-supervised ViT, called ViT-CoT (Vision Transformer with Contrastive Learning through Time) learns by leveraging the temporal structure of natural visual experience, using the time-based contrastive learning objective (Fig 9A). Like the CNN variant, ViT-CoT learns without image labels and without artificial image augmentations.

We tested four different architecture sizes (ViTs with 1, 3, 6, or 9 blocks and attention heads, see Methods). We first examined the untrained performance of ViTs. Unlike CNNs, where untrained models perform better with larger architecture sizes (Fig 5B), all untrained ViT architecture sizes performed roughly the same (Fig 9C, see Methods).

We then examined trained performance. Despite being generic learners, the ViT-CoT models learned to solve the view-invariant recognition task when trained in the same visual environments as newborn chicks (Fig 9B). We observed reasonably strong performance across all architecture sizes and rearing conditions (Fig 9B; one-sample t-tests for each ViT-CoT size and rearing condition combination: all Ps < .05). To quantify the impact of learning, we compared untrained to trained models (Fig 9C). Trained ViT-CoT models showed large learning gains over untrained models (Welch two-sample t-tests comparing untrained and trained ViT-CoTs for each architecture size: all Ps < 10^−7^), matching or exceeding chick-level performance. Thus, ViTs can learn effectively in the impoverished environments faced by chicks.

Despite having the same temporal learning objective, the CNNs performed moderately better than the ViTs, which might be due to the hardcoded spatial knowledge in CNNs, but not ViTs. We emphasize that, while ViTs performed lower than CNNs, the ViTs still succeeded on the task, learning invariant object features. This result shows that generic space-time fitting models can learn view-invariant object recognition in the same environments as newborn animals.

### Behavioral consistency analysis

**Fig 10:**
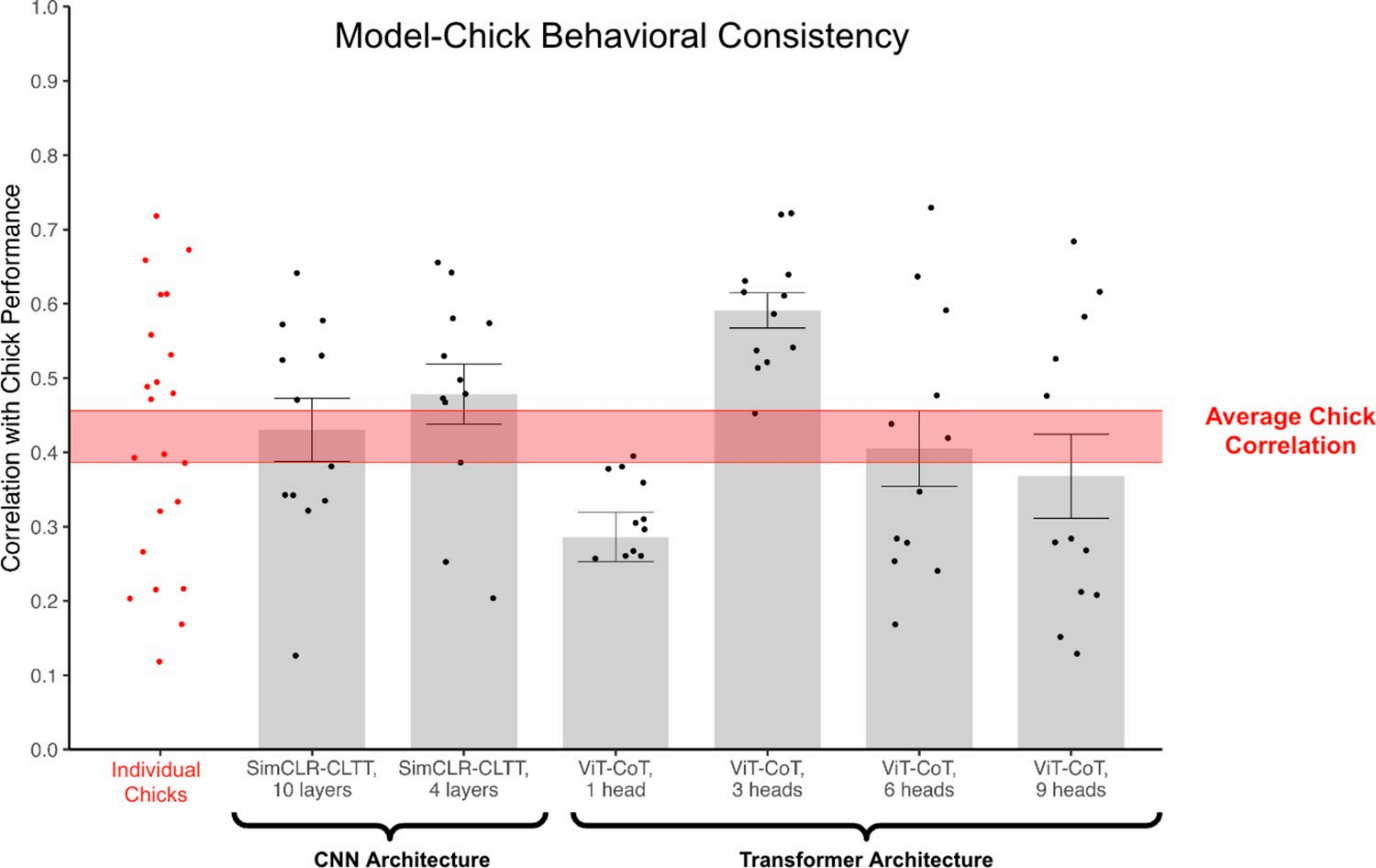
Behavioral Consistency Analysis. Representational similarity between the chicks and models. We measured representational similarity as the correlation between each model’s performance across the 12 test viewpoints and average chick performance across the 12 test viewpoints. We show each chick’s correlation to average chick performance (red dots) and each model’s correlation to average chick performance (black dots). Average and standard error for each model architecture are shown as bars and error bars, respectively. The lower and upper bounds of the chicks’ average correlation are shown as red lines with shading in between. The upper bound shows the mean correlation between each chick and the group-averaged performance across viewpoints. The lower bound shows the mean correlation between each chick and the remaining chicks’ group-averaged performance across viewpoints. The chicks and models generally showed the same pattern of successes and failures across the test viewpoints.

Space-time fitting models can learn to solve the same view-invariant object recognition task as newborn chicks; however, the models and chicks might have used different strategies or features to solve the task. If so, then the chicks and models should show different patterns of successes and failures across the 12 test viewpoints. We first measured whether the pattern of performance across the test viewpoints was reliable across the chicks. To do so, we computed the split-half reliability (with Spearman-Brown correction). Since there are many ways to split the chicks into two groups (each producing a unique reliability estimate), we generated 100 random half-splits and computed the split-half reliability for each split. Reliability across the splits was high (M = 0.84, SD = .07), indicating high internal consistency in performance across chicks. Next, to measure the similarity of chick and model performance, we performed behavioral consistency analyses (Fig 10). Specifically, we calculated the Pearson correlation between each model’s performance across the 12 test viewpoints and the chicks’ performance across the 12 test viewpoints (Methods). We found that all of the space-time fitting models (other than ViT-CoT with one attention head) reached or exceeded the average chick correlation. Thus, space-time fitting models show the same pattern of successes and failures across the test viewpoints as the chicks.

### Unsupervised two-alternative forced-choice evaluation

In the experiments reported above, the space-time fitting models were trained in a self-supervised manner, but we used a supervised linear classifier (decoder) to evaluate the features learned by the models. Can space-time fitting models succeed on this task even when the decoder is self-supervised? After all, the chicks had no supervision during any part of the experiment.

To test this, we used a self-supervised decoder to evaluate the CNNs (Experiment 3) and ViTs (Experiment 4). We used a variation of the technique described by Ayzenberg & Lourenco [100], initially developed to compare machine learning models to human babies. The chicks’ behavior in Wood [42] can be conceptualized as a measure of alignment between the test stimuli and the chick’s internal representation of their imprinted object. Given a choice between two stimuli, chicks will approach the stimulus they perceive to be the most aligned with their representation of the imprinted object. To approximate this in silico, we converted each trained CNN and ViT from Experiments 3–4 into an autoencoder, then tested the autoencoders on the two-alternative forced-choice (2AFC) task presented to the chicks.

We converted the models into autoencoders by attaching a simple fully connected downstream decoder to the (trained and frozen) CNN or ViT encoder (Methods). Then, we performed unsupervised training on the decoder, using the same images that were used to train the encoder. Consequently, both the encoder and decoder were trained only on images of one object shown from one viewpoint range, akin to the chicks. Once the decoder was trained, we used the output from the decoder to quantify how similar each test stimulus was to the model’s representation of the imprinted object.

To mimic the 2AFC task, we fed object images into the autoencoder (i.e., encoder + decoder), then measured the reconstruction error for each image. If the model successfully ‘recognized’ the imprinted object, then the average reconstruction error should have been smaller for the imprinted object than the novel object. We evaluated the models across all 12 of the viewpoint ranges.

We found that the reconstruction error was higher for the novel object than the imprinted object for the SimCLR-CLTT CNN model (Welch’s independent samples t-test, t(4783.1) = 9.0, p < 10^−15^) and for three of the four the ViT-CoT architectures (Welch’s independent samples t-tests, 1-layer: t(4790.3) = 6.1, p < 10^−9^, 3-layers: t(4796) = 8.0, p < 10^−14^, 6-layers: t(4797.4) = 7.5, p < 10^−13^, 9-layers: t(2399.2) = 1.4, p = .15). Thus, a fully self-supervised generic temporal learning model, in which both the encoder and decoder are trained without any supervised signals, can learn to solve the same view-invariant recognition task as newborn chicks.

### Experiment 5: Comparing models trained in natural visual worlds versus controlled-rearing chambers

Experiment 4 suggests that the visual information available in a simple white rectangular room—containing a single object shown from a limited 60° viewpoint range—is sufficient for a generic space-time fitting model to learn invariant object features. This finding may seem counterintuitive, given that 1) the model started with no hardcoded knowledge about objects or space, and 2) the environments were impoverished compared to natural environments. But, one powerful feature of image-computable models is that scientists no longer need to rely on their intuitions about what is learnable and what is not from particular visual experiences. Instead, scientists can directly test whether a model learns better in some environments than others.

Classic views of visual development have tended to focus on objects, surfaces, textures, and scenes as the units of visual learning. Alternatively, brains might learn more like direct-fit models: By acquiring large numbers of unique views of an environment, direct-fit models can learn to approximate the distal data distributions producing proximal retinal images. Learning the data distributions underlying a three-dimensional (3D) environment from a collection of views requires implicitly learning how 3D shape changes across views. From this perspective, even impoverished 3D environments, like those used in the chick experiments, may provide sufficient information for learning object shape. If so, then direct-fit models trained in controlled-rearing environments might approach the object recognition performance of models trained in natural environments.

**Fig 11:**
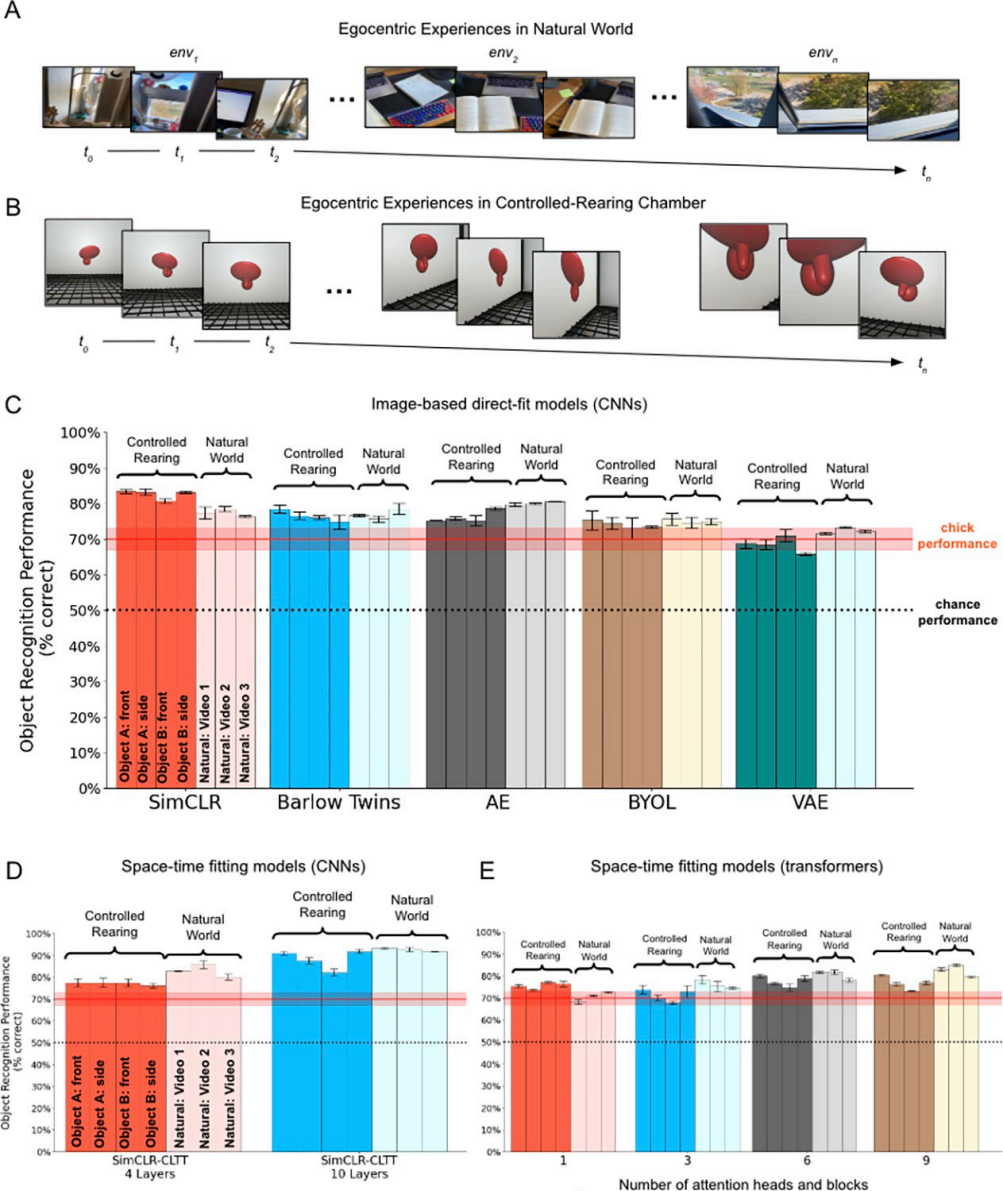
Comparison of deep neural networks trained in **(A)** natural visual environments versus **(B)** controlled-rearing chambers. **(C)** View-invariant recognition performance of the image-based CNN models from Experiment 1. **(D)** View-invariant recognition performance of the CNN space-time fitting models from Experiment 3. **(E)** View-invariant recognition performance of the transformer space-time fitting models from Experiment 4. Models trained in natural environments generally performed at similar levels as models trained in controlled-rearing chambers. Error bars represent standard error of model performances across validation folds. The sample images shown in panel A are for illustrative purposes only and were not used for training the models.

To test this possibility, we repeated many of the experiments described above, except we changed the training data (Fig 11): rather than training models in the controlled-rearing chambers, we trained the models with head-mounted camera data from human adults performing a wide range of activities in natural environments (UT Ego Dataset, [101]). We then compared the object recognition performance of these naturally trained models with the models trained in the controlled-rearing chambers. The naturally trained models (Fig 11A) and controlled-rearing trained models (Fig 11B) received the same number of training images and were tested on the same view-invariant object recognition task used in Experiments 1–4.

Across the image-based CNN models from Experiments 1–2 (Fig 11C), the space-time fitting CNN models from Experiment 3 (Fig 11D), and the space-time fitting ViT models from Experiment 4 (Fig 11E), the models trained in the controlled-rearing chambers performed at similar levels as the models trained in natural environments. While some models performed better when trained with the UT Ego Dataset (Welch Independent Samples t-tests: Autoencoder: t(13.8) = 6.8, p < 10^−5^; VAE: t(14.2) = 4.5, p = .0004, SimCLR-CLTT 4-layers: t(15.6) = 4.4, p = .0005, SimCLR-CLTT 10 layers: t(12.8) = 3.4, p = .004, ViT-CoT 3-head: t(18.5) = 3.2, p = .005, ViT-CoT 6-head: t(18.9) = 2.8, p = .01, ViT-CoT 9-head: t(18.3) = 4.8, p = .0001), other models performed better with the controlled-rearing dataset (SimCLR: t(15.0) = 6.8, p < 10^−5^, ViT-CoT 1-head: t(15.9) = 5.6, p < 10^−4^), and other models showed no difference (Barlow Twins: t(18.5) = 0.5, p = .61; BYOL: t(18.8) = 0.8, p = .43). For direct-fit models, learning to solve this view-invariant object recognition task does not require a natural visual diet filled with large numbers of objects, surfaces, textures, and scenes. Rather, direct-fit models learn object recognition by leveraging large numbers of views (Fig 6B).

We see this experiment as a starting point for addressing a core question at the heart of developmental psychology: Which experiences matter for learning object perception? Future studies might perform in silico controlled-rearing experiments on DNNs, systematically ablating different features in the training data to explore which features matter—and which do not—for learning object recognition. Our digital twin method provides the foundation for a rigorous exploration of the role of experience in the development of object perception, through parallel experiments of newborn animals and computational models.

## Discussion

We compared learning across biological and artificial visual systems by performing parallel controlled-rearing experiments on newborn chicks and deep neural networks. Using digital twins, we trained the chicks and models in the same visual environments and tested their object recognition performance with the same stimuli and tasks. We found that direct-fit models (CNNs and transformers) learn invariant object recognition when trained in the same environments as chicks. We also found that a particular class of direct-fit models—space-time fitters—learn invariant object recognition by using time as a teaching signal, akin to biological visual systems. Like chicks [11–13,96], space-time fitting models learn object representations by adapting to the spatiotemporal data distributions in the environment. Our results provide methodological and theoretical contributions for studying the origins of vision.

### Methodological contributions

A core scientific goal is to build working computational models of brains. However, three challenges have prevented scientists from building image-computable models of newborn visual systems. First, researchers lacked controlled-rearing methods for obtaining precise data from newborn subjects, preventing reliable measurement of how vision changes as a function of experience. Second, researchers lacked scalable computational models that could learn from raw visual inputs in an unsupervised manner, akin to animals. Third, researchers lacked a platform for simulating the visual experiences of newborn animals. Due to these challenges, current theories of visual development are not image computable, requiring a human in the loop to determine what prediction a theory should make in response to particular visual experiences.

We present solutions to all three challenges: (1) automated controlled rearing solves the first challenge, allowing researchers to collect precise measurements of how an animal’s behavior changes as a function of particular visual experiences [12,51,55,96]; (2) self-supervised DNNs solve the second challenge, allowing researchers to build image-computable models that learn from raw visual experiences; and (3) video game engines solve the third challenge, offering a platform for simulating the visual experiences of newborn animals in realistic environments. These solutions provide a foundation for building image-computable models of newborn visual systems (i.e., models that learn in the same visual environments as newborn animals).

How does the number of images used to train models compare to the number of images that shape newborn visual systems? The field does not have well established procedures for comparing the number of training images across animals and DNNs, which is why we focused on controlling the visual environment available to chicks and models, rather than controlling the number of training images per se. However, to make a rough comparison, researchers have suggested that biological visual systems carry out a form of iterative, predictive error-driven learning every 100 ms, which corresponds to the widely studied alpha frequency of 10 Hz originating from deep cortical layers [102]. If each 100-ms learning window is thought of as a single training image in a computer vision task, then newborn animals acquire ~36,000 training images in the first hour after birth (10 images/sec × 60 sec/min × 60 min/hr). If newborns spend half their time sleeping, then they will still acquire ~430,000 images in their first day and ~3 million images in their first week. In this context, the 80,000 images we used here amounts to ~4 hours of visual experience (which is much less than the chicks received).

Consequently, while newborns might not have access to data streams with large numbers of objects or environments, they do have access to data streams with large numbers of views. During everyday experience, newborns engage in self-generated data augmentation, acquiring large numbers of unique views from diverse body positions and orientations. Our results show that direct-fit models—such as space-time fitters—can leverage large numbers of views to learn invariant object features in impoverished environments. For both chicks and space-time fitting models, environments with a single object contain sufficient data to learn invariant object recognition.

We largely focused on CNNs because they share characteristics with newborn visual systems; both CNNs and newborn visual systems have a hierarchical and retinotopic organization [28,103]. Moreover, the CNN receptive field structure could plausibly emerge during prenatal development. Generic learning models (e.g, fully connected networks, transformers) can learn a convolutional organization when trained on data with non-Gaussian, higher-order local structure, resulting in the localized, space-tiling receptive fields that characterize CNNs [104,105]. During prenatal development, visual systems are shaped by internally generated signals (spontaneous retinal waves) with non-Gaussian, higher-order local structure. Thus, prenatal processes could grow CNN-like receptive fields from more generic (transformer-like) learning machinery. Support for this hypothesis comes from simulations showing that CNN-like networks can be grown from a single cell using two ingredients present during prenatal development: 1) spontaneous retinal waves and 2) spike-timing-dependent plasticity [106,107]. We speculate that generic temporal learning machinery (generic space-time fitting) underlies vision, and that prenatal training data (retinal waves) shape this machinery into a CNN-like hierarchical and retinotopic architecture by birth. The resulting CNN-like architecture then scaffolds and constrains subsequent postnatal learning.

### Theoretical contributions

Our simulations suggest that direct-fit learning [20] is a viable strategy for learning how to see. Unlike classic theories in psychology, direct-fit models do not learn simple rules and representations. Rather, direct-fit models learn complex, high-dimensional representations by iteratively adjusting large numbers of parameters in order to adapt (fit) to the structure of the data. With sufficient data, the representations learned by direct-fit models will approximate the distal variables (e.g., objects, scenes) that produce proximal retinal images [82]. We emphasize that although the models that implement direct fit are complex (e.g., millions of adjustable parameters in CNNs and ViTs), direct-fit models are conceptually simple and parsimonious. In fact, the fitting process mirrors the fitting processes driving natural selection, in which organisms become adapted to their environment through iterative selection (see ref. [20] for extended discussion).

In particular, we suggest that visual systems are space-time fitters, meaning visual development can be understood as a blind fitting process in which visual systems gradually adapt to the spatiotemporal data distributions in the newborn’s environment. The core assumption underlying this view is that visual intelligence emerges from a generic temporal learning system. To fit to the environment, the visual system learns from space-time transitions between views. We hypothesize that as space-time fitting models learn (fit) to prenatal and postnatal visual environments, they will gradually develop the visual skills found in young humans and animals. Our results provide an existence proof that space-time fitting models can learn common visual skills as newborn animals when trained in the same environments as animals.

The benefit of thinking about visual development in terms of fitting, rather than learning more generally, is that fitting provides a concrete definition of learning. The term learning can refer to a wide range of different processes, and attempts to define learning often do not provide concrete predictions. For example, a common definition of learning is “functional changes that result from experiences” [112], but this type of definition has been criticized as being too broad to guide research in developmental psychology [113]. Conceptualizing learning as fitting helps solve this problem because fitting processes can be instantiated in image-computable learning models (e.g., deep neural networks). These models can then be run in simulations to determine what they learn when fitting to a particular environment. Thus, fitting models produce precise, image-computable predictions for studying visual learning. This is especially true when fitting models are combined with controlled-rearing methods: Researchers can ‘rear’ fitting models and newborn animals in the same environment to directly measure whether they learn the same capacities when fitting to the same environment.

Ultimately, studying visual development (and development more generally) within a fitting framework would connect developmental psychology to a much larger body of research. Researchers across biology, anthropology, and sociology use fitting principles to understand the evolution of animal species and cultural knowledge. Both biological and cultural evolution are conceptualized in terms of general fitting principles: variation + selection optimization. Variation generates a range of possibilities, and selection filters those possibilities based on fitness to an objective. These same fitting principles underlie direct-fit models, which start with random weights (variation) followed by gradual adjustment of those weights (selection). By training direct-fit models in the same environments as newborn animals, we have shown that visual development can be understood in terms of fitting principles. This view could help unify fields, with evolution, culture, and development all studied under a common fitting framework, with shared general principles.

Finally, our results suggest that Wood’s [42] original conclusion needs revision. Based on the chicks’ rapid learning, Wood argued that “…powerful, robust, and invariant object recognition machinery is an inherent feature of the newborn brain.” But, our simulations show that this task is readily learnable from the visual diet available to chicks, so it is not necessary to postulate the existence of innate invariant object features to explain the chicks’ behavior. We now argue that “…powerful, robust, and generic temporal learning machinery is an inherent feature of the newborn brain; this machinery is sufficient to learn invariant object features from the visual experiences available to newborn animals.”

### Future directions and limitations

By discovering self-supervised models that can solve the same object recognition tasks as newborn animals, these results set the stage for exciting future directions. We can now search through the direct-fit model class to find particularly strong models, via a continuous cycle of model creation, model prediction, and model testing against new experimental results. Over time, we can cull models that are less accurate and focus attention on improving and extending the most accurate models. Controlled comparisons with different architectures, objective functions, and learning rules could define the necessary and sufficient learning mechanisms for newborn-like visual processing [98]. Controlled comparisons using the same learning machinery, but different training data (e.g., from different controlled-rearing experiments), could also reveal which visual experiences are necessary and sufficient to develop visual intelligence.

In the present study, we created a landscape of models that systematically varied in terms of learning algorithm, architecture size, architecture type, number of training images, and number of views used to train the linear classifier. We observed systematic patterns of change across these dimensions, showing that some attributes are better than others for learning invariant features in impoverished environments (e.g., a contrastive learning algorithm, dense sampling of the visual world, CNN architecture, and more views to train downstream linear classifiers). Future experiments could also systematically increase the difficulty of the task to provide more fine-grained benchmark data from newborn animals.

One limitation of the current study is that we tested models across one animal study (four rearing conditions). Future experiments could test models across a wider range of studies, adopting the integrative benchmarking approach used in computational neuroscience [32]. Automated controlled-rearing studies have revealed systematic patterns of successes and failures in the visual learning skills of newborn chicks [11–13,51,52,96]. By testing whether chicks and models show common patterns of successes and failures across a wide range of studies, the field could discover learning algorithms that mimic the learning mechanisms in newborn brains.

A second limitation is that we trained models passively. This contrasts with the active learning of newborn animals, who interact with their environment to produce their own training data [23]. By choosing where to go and what to look at next, biological systems generate their own curriculum to suit their current pedagogical needs [54]. Future studies could close this gap between animals and machines by embodying DNNs in artificial agents that collect their own training data from the environment. We have made initial steps in this direction, by releasing benchmarks that allow researchers to train and test embodied DNNs in virtual controlled-rearing chambers that mimic the controlled-rearing chambers used to raise and test newborn chicks [109,110].

Future research could also more closely match the visual diet across animals and models. While we largely closed this gap by simulating the first-person images obtained by agents moving through environments that mimicked those faced by chicks, we did not capture the exact visual data acquired by chicks. Researchers might fine-tune this digital twin approach by yoking the camera in the virtual environment to the chick’s head in the real environment, thereby ensuring that the camera collects the same visual images as the chicks. Likewise, our study controlled the postnatal experiences provided to newborn chicks and models, but newborn visual systems also learn from prenatal visual experiences (e.g., spontaneous retinal waves; [108]). In principle, retinal waves might provide sufficient training data for learning some object perception skills. Future studies might pretrain DNNs on simulated retinal waves, giving animals and models access to the same initial prenatal experiences.

A third limitation is that although we show that direct-fit models can learn to solve the same tasks as newborn chicks from the same visual diet, we do not know the extent of algorithmic equivalence between the models and chicks. Future studies could extend this digital twin approach by exploring whether chicks and models respond to similar stimulus features, using well-established techniques from vision science [111]. Our results also do not imply that chicks or models learn complex 3D geometric representations of whole objects [62]. They could learn to solve invariant object recognition tasks by learning invariant representations of subfeatures that are smaller than the entire object. These feature detectors might respond to only a portion of the object, or be sensitive to key 2D, rather than 3D, features [63]. Many computational models of object recognition in humans and animals rely on such subfeatures. Regardless of the nature of these features, they allow models to succeed on a visual recognition task that requires recognizing objects across large, novel, and complex changes in an object’s appearance: the hallmark of invariant object recognition [62–64]. Our paper shows that these features—which are tolerant to image-level variation due to changes in view—are learnable from the visual experiences available to newborn chicks. It will be interesting for future studies to examine the specific nature of the features learned by chicks and models.

## Conclusion

We present evidence for parallel development of object recognition in newborn chicks and deep neural networks. Like chicks, the models learned invariant object features from raw visual experiences in impoverished environments, permitting recognition of familiar objects across large, novel, and complex changes in the object’s appearance. One class of direct-fit models—space-time fitters—can even learn object recognition when equipped with a biologically plausible learning objective that leverages time as a teaching signal. Our findings lay a foundation for linking controlled-rearing studies of newborn animals to image-computable models from artificial intelligence. This digital twin approach extends the reverse-engineering framework pioneered in computational neuroscience to the study of newborn vision, supporting the broader goal of building unified models of the learning machinery in brains.

## Methods & materials

### Design of animal experiment

The behavioral data were originally reported in Wood [42]. The study tested 35 newborn chicks of unknown sex. Wood [42] obtained the eggs from a local distributor and incubated them in darkness. After hatching, the chicks were transferred to controlled-rearing chambers in darkness, with the aid of night vision goggles. Each chick was reared separately in its own chamber.

The controlled-rearing chambers were constructed from white, high-density plastic and measured 66 cm (length) by 42 cm (width) by 69 cm (height). The chambers had wire mesh floors supported 2.7 cm off the ground by transparent beams. Transparent holes in the floor held food and water. To track the chicks’ behavior, Wood [42] used microcameras and image-based tracking software to measure the amount of time that each chick spent in zones next to the left and right display walls (i.e., LCD monitors).

During the experiment, the controlled-rearing chambers displayed virtual objects that measured 8 cm in length by 7 cm in height, suspended 3 cm off the floor. The objects rotated through a 60° viewpoint range about an axis passing through its centroid, completing the full back and forth rotation every 6s. The objects were displayed on uniform white backgrounds at the center of the display walls.

Fig 2 shows the design and stimuli used in the chick study. During the training phase (first week), the imprinted object was presented from a single 60° viewpoint range. The object appeared on one display wall at a time, appearing an equal amount of time on the left and right display walls. Half of the chicks were imprinted to object A (with object B serving as the unfamiliar object), and the other half of the chicks were imprinted to object B (with object A serving as the unfamiliar object). During the test phase, the chicks received one 20-min test trial every hour. In each test trial, the imprinted object appeared on one display wall (from a familiar or novel viewpoint) and the unfamiliar object appeared on the other display wall. We tested 12 viewpoint ranges (11 novel, 1 familiar) 14 times within randomized blocks over the course of the test phase. Videos 1–4 also show simulated first-person images during the training phase and test phase. https://github.com/buildingamind/ChicksAndDNNs_ViewInvariance.git

Wood [42] created the 12 viewpoint ranges by rotating the objects 360° degrees around four axes: frontoparallel vertical axis, frontoparallel horizontal axis, a frontoparallel vertical axis tilted +45°, and a frontoparallel vertical axis tilted -45°. Wood [42] cut each 360° image sequence into four 90° segments, then trimmed 15° from the left and right of the segment (to reduce overlap across viewpoint ranges), resulting in the final 60° viewpoint range. Wood [42] then removed four viewpoint ranges because they partially overlapped with other viewpoint ranges (e.g., at the starting position where all four 360° rotations overlapped).

### Design of digital twin experiments (General Methods)

The virtual animal chambers were created in the Unity game engine. We designed the virtual chambers to be as similar as possible to the real-world chambers from Wood [42]. The virtual chambers had the same proportions as the animal chambers, with alternating display walls and white walls. The display walls mimicked the 19” LCD displays that presented the virtual objects in the animal chambers. This digital twin environment allowed us to simulate the training data (visual experiences) available to the chicks with near photorealistic accuracy.

### Stimuli generation

We used the virtual chambers to simulate the training data available to the chicks. As in the chick studies, we projected the same object stimuli on the display walls in the virtual chambers. We created a virtual agent (3.5 units height × 1.2 units length) with an invisible forward-facing camera attached to its head to collect visual observations. We then recorded the first-person images (64×64 pixel resolution images) acquired by the agent moving through the chamber. To canvas the visual experiences available in the chamber, we programmed the agent to collect visual experiences in the following manner (see Videos 1–2 for examples at https://github.com/buildingamind/ChicksAndDNNs_ViewInvariance.git). First, the agent picked a random location inside the chamber and gradually moved to that location, at the rate of 1.5 units/s. During this motion across the chamber, the agent kept its gaze centered on the object projected on the display wall. Once the agent reached the location, the agent moved its head 30° in each of the directions (negative and positive) of the three axes of rotation (6 rotations in total, order chosen randomly). The agent performed the head rotations along the three axes in ~9.5 sec. The agent then picked a new random location in the environment and repeated the cycle.

Since the controlled-rearing chambers were asymmetric (i.e., the food and water were located on one side of the chamber), we collected half of the training images when the imprinted object was on the right display wall and half of the training images when the object was on the left display wall. We collected 80,000 training images from the agent for each of the four rearing conditions described in Wood [42] (Fig 2A). Like the chicks, each model was only trained in one of the four rearing conditions. For each model configuration in each of the four rearing conditions, we trained three models (each with a different random seed), yielding 12 models per model class.

We used the same simulation approach described above to collect the test images, except that we projected the test videos (rather than the training videos) on the display walls (see Videos 3–4 for examples at https://github.com/buildingamind/ChicksAndDNNs_ViewInvariance.git). We collected 11,000 test images from each of the 12 viewpoint conditions presented to the chicks. We then used these test images to train and test the linear classifiers.

Below, we present methodological details specific to each experiment.

### Experiment 1

#### Neural network architectures

As a starting point, we used a standard ResNet-18 architecture. A standard ResNet-18 architecture contains four residual blocks, with each residual block having two basic blocks. Each basic block contains hidden layers (convolutional and pooling operations).

### Learning algorithms

As discussed in the main text, we evaluated several self-supervised learning algorithms (Table 1): autoencoders [77], variational autoencoders [78], contrastive embedding methods (SimCLR [69]), asymmetric networks (BYOL [70]), joint embedding learning (Barlow Twins [68]), and GreedyInfoMax [92]. These algorithms learn features in different ways. Autoencoders project input to a lower-dimensional latent embedding space, producing a compressed representation of the training data. To learn the latent representation, autoencoders reconstruct the inputs in the original, higher-dimensional space and tune the model weights to produce the best reconstructions. An input can be encoded either as a single point in the latent space (autoencoder) or as a distribution over the latent space (variational autoencoder). Contrastive embedding methods learn features by mapping different augmented versions of an image close to one another in the latent space. In SimCLR, positive image pairs are generated by applying random transforms (e.g., image blur, color jitters) to an image in the training batch. The rest of the images in the batch are treated as negative examples and mapped far away from the positive image pair in the latent space. Asymmetric network methods (e.g., BYOL) use two neural networks to produce low-dimensional embedding spaces: a target network that is updated slowly and an online network that is trained to predict the target network’s representation of augmented image views. Joint embedding architectures (e.g., Barlow Twins) learn by training two neural networks to produce similar embeddings for different views of the same image, producing visual representations that are invariant to changes in an object’s appearance. Finally, GreedyInfoMax learns without backpropagation by maximizing the mutual information inputted to, and outputted from, each layer in the model. GreedyInfoMax’s layer-by-layer learning approach optimizes an InfoNCE within each layer, without any backward flow of gradients.

**Table 1:** Hyperparameters of self-supervised learning algorithms used in Experiments 1–5.

Model	Learning Objective	Encoder Type	Batch Size	Epochs	Output Dimensions	Warm-up Epochs	Image Augmentations
SimCLR	Contrastive Learning	CNN	512	100	512	5	Yes
BYOL	Asymmetric Embedding	Siamese CNN	512	100	512	10	Yes
Barlow Twins	Joint Embedding	Siamese CNN	512	100	512	10	NA
VAE	Image reconstruction	CNN	128	100	512	0	NA
AE	Image reconstruction	CNN	128	100	512	0	NA
GIM	Contrastive Learning	CNN	32	100	512	0	Yes
SimCLR- CLTT	Contrastive Learning Through Time	CNN	512	100	512	5	NA
ViT-CoT	Contrastive Learning Through Time	Transformer	128	100	512	0	NA

The hyperparameters used in all the models were chosen based on the default and optimal values reported in the original studies [68–70,77,78,92,97,98]. All of the self-supervised models in Experiment 1 were trained on 10,000 images, except for GreedyInfoMax, where each model was trained on 80,000 images.

### Linear classifiers

After training the CNNs using the self-supervised learning algorithms, we evaluated the classification performance of the CNNs using the test stimuli (see Stimuli Generation, above). Task performance was assessed by removing the last fully connected layer of the network, adding a new fully connected linear readout layer on top of the last layer of each trained CNN encoder, and then training only the parameters of the readout layer on the binary object classification task. The linear classifiers contained 512 input neurons, each of which received input from one of the 512 neurons in the final layer of the CNNs. The linear readout layers were optimized for binary cross-entropy loss.

To train and test the linear classifiers, we used the test images collected from the agents moving through the virtual chambers (11,000 images for each of two objects across 12 viewpoint ranges). When training the linear classifiers, the object identities were used as the ground-truth labels. Since the CNN weights were frozen, the supervised training of the linear classifiers did not change the features learned by the CNNs.

To evaluate whether the features learned by the CNNs could generalize across novel viewpoints, we used a cross-validated K-fold analysis to train/test the linear classifiers, where each fold contained images from one of the 12 viewpoint ranges. We used two different training and test splits. The first split technique used the traditional K-fold splits that are standard across the field. The second split technique inverted the traditional K-fold splits to reflect the limited available training data for the chicks. We describe each below:

- **Traditional K-Fold Splits, N**_**train**_ **= 11; N**_**test**_ **= 1:** The test images were divided into 12 folds, with each fold containing images of each object rotating through 1 viewpoint range. The linear classifiers were cross-validated by training on 11 folds (11 viewpoint ranges) and testing on the held-out fold (1 viewpoint range).
- **Single Training View Splits, N**_**train**_ **= 1; N**_**test**_ **= 11:** The linear classifiers were cross-validated by training on 1 fold (1 viewpoint range) and testing on the 11 held-out folds (11 viewpoint ranges). In this extreme case, the linear classifiers were trained using only 1 viewpoint range from each object and tested on the remaining 11 viewpoint ranges.

In all of the train-test splits, the linear classifiers were trained on 11,000 total images. During training, we used a batch size of 128 for 100 epochs. For each linear classifier condition, transfer performance was evaluated by first fitting the parameters of the linear classifier on the training set and then measuring classification accuracy on the held-out test set. We report average cross-validated performance on the held-out images not used to train the linear readout layer. Thus, all of our results reflect the generalization performance of the models across novel viewpoints.

### Experiment 2

As shown in Table 2, we systematically manipulated the size of the architectures by changing (a) the number of residual blocks and (b) the number of hidden layers in the basic blocks. These architectures were then trained using the self-supervised learning algorithms described below. Each combination of architecture and self-supervised algorithm was trained with 3 different seeds (i.e., specific random initialization of weights) per rearing condition. Following Chen et al. [69], we used linear warmup for the first 5 epochs when training SimCLR and the first 10 epochs when training BYOL and Barlow Twins.

**Table 2:** ResNet with different architecture sizes.

Architecture	Residual Block(s)	Hidden Layers Per Residual Block	Total Hidden Layers*	Parameters
ResNet34	4	7, 8, 12, 6	34	21.5M
ResNet18	4	5, 4, 4, 4	18	11.4M
ResNet14	3	5, 4, 4	14	8.4M
ResNet10	2	5, 4	10	7.6M
ResNet4 (Exp 3)	1	4	4	2.9M

*Total Hidden Layers include a fully connected layer after the residual blocks. For all our experiments, we remove the last fully connected layer to get an output embedding of shape 512.

### Experiment 3

#### Contrastive learning through time algorithm

To train the CNN encoder using time as the teaching signal, we modified the Contrastive Learning Through Time algorithm (SimCLR-CLTT) [69,97]. This algorithm incorporates a temporal window of N samples where the temporal dependency between each consecutive frame is preserved. The features extracted from images within the same temporal window were brought close to one another in the feature space, while simultaneously being pushed away from all other features not in the temporal window. All of the models in Experiment 3 were trained with 80,000 images.


$${lossz}_{t}=‐log\frac{exp(sim(z_{t},z_{t+1})/\tau )+exp(sim(z_{t},z_{t+2})/\tau )}{\sum_{k=1,k\neq t}^{2N}\;exp(sim(z_{t},z_{k})/\tau )}$$ (1)

### Experiment 4

#### Vision transformers

To train the Vision Transformers (ViTs), we used the same objective function—Contrastive Learning through Time (CLTT)—that we used for training the CNNs (see Experiment 3 above for the loss function). We systematically varied the architecture size by adding transformer blocks and attention heads, resulting in four different sizes (1H, 3H, 6H, and 9H) (Table 3). For example, in the case of ViT-CoT(3H), the architecture consisted of three attention heads and three transformer blocks. Each architecture was trained on 80k samples using three different seeds.

**Table 3:** ViT-CoT with different architecture sizes.

Architecture	Attention Head(s)	Transformer Layer(s)	Parameters
ViT-1H	1	1	5.8M
ViT-3H	3	3	16.9M
ViT-6H	6	6	36.4M
ViT-9H	9	9	59.4M

In the training process, patches of 8×8 were created from 64×64 images. These patches were then flattened, and a class token was appended to this flattened vector. Before passing this vector to the encoder, a position embedding was added. In the encoder, each attention head operates on this vector, attending to different features in the image. The concatenated output from each head is then passed to the next transformer layer for processing. For details on implementation, see [98].

### Behavioral consistency analysis

The behavioral consistency analysis (Fig 10) measured the similarity in the pattern of performance across the chicks and models. We first computed the average chick performance for each test viewpoint (across all chicks from Experiments 1 and 2 in Wood, 2013). We then generated an upper and lower bound of the mean correlation between each chick and average chick performance across the viewpoints. To calculate the upper bound, we computed the Pearson correlation between each chick and the average chick performance across the viewpoints. The upper bound was the average of those Pearson correlations; however, this bound can be inflated because each chick’s performance is included when calculating the average, artificially boosting the correlations. To calculate the lower bound, we computed a leave-one-out average for each chick. The leave-one-out average was the average chick performance excluding that particular chick. Then, we correlated each chick’s performance with that specific chick’s leave-one-out average. The lower bound of the noise ceiling was the average of those Pearson correlations.

To evaluate the models, we computed the Pearson correlation between each model’s performance across the viewpoints and average chick performance across the viewpoints. This correlation measured the similarity in the pattern of performance across chicks and models (i.e., did chicks and models find the same viewpoints easy or hard?). We then computed the average (and standard error) of those correlations for each model architecture. To allow direct comparison of models and chicks, we show both the average chick correlation (red lines, showing upper and lower bound) and each individual chick’s correlation with average chick performance (red dots).

Thus, our behavioral consistency analysis (Fig 10) contained four elements: (1) each individual chick’s correlation with average chick performance across the viewpoints (red dots), (2) the average of those chick correlations, with upper and lower bounds (red lines), (3) each individual model’s correlation with average chick performance (black dots), and (4) the average correlation for each model architecture (gray bars).

If a model shows a different pattern of performance than chicks, then the model’s correlation with chicks should be lower than the average chick correlation. Conversely, if a model produces the same pattern of performance as chicks (with similar variability as chicks), then the model’s correlation with chicks should be similar to the average chick correlation. Finally, if a model produces the same pattern of performance as chicks (but with lower variability than chicks), then the model’s correlation with the chicks should be higher than the average chick correlation.

### Unsupervised classification via reconstruction loss

Each CNN model was transformed into an autoencoder by adding a two-layer fully connected decoder. The encoder was subsequently frozen, and the decoder was trained on the same samples used to train the encoder. The autoencoder was trained with a batch size of 128 for 100 epochs. Mean Squared Error (MSE) loss objective was used to train the decoder network of the model. Once the decoder was trained, we passed all of the test images through the autoencoder and compared the reconstruction loss between the images of the imprinted object and the images of the unfamiliar object using independent-samples *t*-tests.

### Experiment 5

#### UT Ego dataset

We used one of the publicly available datasets from UT Austin Egocentric (UT Ego) [101]. The data were recorded using a head-mounted camera worn by three different graduate students. Our models were trained on one of the three videos. For each model/video combination, we trained three randomly initialized seeds. The head-mounted data captured the students’ daily activities across various environments. Each video was approximately 4 hours long with a resolution of 480x320.

### Statistical analysis

We used the R programming language and tidyverse package to investigate the performance of different neural network architectures, learning objectives, and amounts of training data. To compute mean performance and standard error for a specific model architecture, we first treated each instance of the model architecture (i.e., each model generated from its own random seed) as a “subject.” By calculating the average performance for each subject, we obtained the "subject-level" performance, which allowed us to compute the average and standard error (N = 3) for the model architecture.

We used Python to visualize 2-D projections of the feature representations of our models using both LDA and t-SNE. LDA is a statistical method that reduces dimensionality by finding a linear combination of features that maximizes the separation between classes while minimizing the within-class variance. For our classes, we used the ground truth labels of whether an image showed Object A or Object B. Unlike LDA, t-SNE is fully self-supervised and requires no labels for dimensionality reduction. t-SNE uses probability distributions to model pairwise similarities between data points, emphasizing the preservation of local structures and clustering patterns.
